# Phthisis

**Published:** 1835-07-01

**Authors:** 


### Medico-Chirurgical Review,

### No. XLV.

**[No. 5 of a Decennial Series.]**

APRIL 1, TO JULY 1, 1835.

1. Pathological Researches on Phthisis. By *E. C. A. Louis,* M.D. &c. Translated by *Dr. Cowan.* Octavo, pp. 338. 1835.

2. A Treatise on Tubercular Phthisis. By *James Clark,* M.D. F.R.S. (From the Cyclopædia of Practical Medicine.) 1834.

3. Consumption, why so Fatal? &c. By *John Tyrrell.* 1835.

The three works whose titles are affixed to this article, are recommended to our favourable notice by characteristic and peculiar merits; merits however differing not less in kind than in degree. The first is distinguished by the originality and comprehensiveness of its views, and by the rare accuracy of its details; the second by the perspicuity of its descriptions, and the practical sound sense of its precepts; and that of John Tyrrell, Gent. by being the production of an amateur Medicus, who has boldly stepped forward to enlighten our profession, and the public at large, on the true nature and proper treatment of a disease, which, he affirms, "is confessedly as little understood now, as it was 2300 years before the discovery of the circulation." Now, although we do not quite agree with Mr. Tyrrell, as to the truth of this most humiliating announcement, and although we do not anticipate much benefit in the treatment of pulmonary consumption, from the remedial means which he has proposed (practising deep inspirations and expirations of cool air, for the purpose of distending the bronchial tubes and pulmonary vesicles, and thus mechanically removing the congested state of the lungs in the neighbourhood of any tuberculous portion), we are willing, and happy too, to award to him the meed of praise which the humanity of his motives, and the industry of his researches, very fairly entitle him to. Let him not "be deterred by the dread of freezing criticism, nor by the frown of power and hiss of bigotry." Critics are not the freezing, frowning, hissing monsters which his imagination has conjured up. By us, at least, he shall be gently treated, and even encouraged to persevere in his laudable emprise of medical authorship, as it may afford a pleasing pastime to himself, and will almost certainly exalt his reputation in the circle of his acquaintances; but, as sincere and *disinterested* friends of our lay brethren, we earnestly advise Mr. Tyrrell to eschew the actual treatment of consumption, either in his own case, or in that of any of his friends, and to confine his researches to the more intellectual task of reading and writing on the physiology, pathology, and we do not even object to, the literary therapeutics of the disease.

We invite him, and all our other courteous readers, to follow us in the analytical review which we now propose to give of the Pathological Researches of M. Louis, and of certain portions of Dr. Clark's treatise; for sure we are, that a studious acquaintance with these two excellent works will powerfully contribute to a more correct knowledge of the causes, symptoms, and effects of pulmonary consumption, and will thus lead the medical practitioner to a more rational, and, we may hope, a more successful mode of treating it. Both authors agree in referring the primary and original cause of the disease to a peculiar cachexy, or constitutional unhealthiness, which has been described under various names, such as the scrofulous or strumous diathesis, latent scrofula, the tubercular cachexia, the morbidly-lymphatic temperament, &c. and one of whose characters is a tendency to the deposition of the peculiar matter called tuberculous, in various organs and tissues of the body. To this conclusion they have arrived by very different roads; Louis, by the results of his minute pathological investigations of the changes left after death by the disease—Dr. Clark, by attentively examining its living features, and, as it were, its outward physiognomy, as revealed in the history of the patient's previous, as well as present constitution and state of health. It is to the former that we shall first and more particularly direct our readers' attention, leaving any remarks on the latter till the close of the article, if our prescribed limits do not then happen to be exceeded.

The labours of the French anatomists during the last thirty years have reaped a rich harvest of practical usefulness, as well as of most honourable fame. It is to them that we owe our present accurate knowledge of most subjects of pathology; and on none can they more justly lay claim to exclusive excellence, than on that which regards the various morbid changes which precede, accompany, and which are induced by phthisis, or pulmonary consumption. Bayle and Laennec laid the foundation and reared the superstructure; Andral and Louis have strengthened the one, and have completed and adorned the other. The peculiar excellences of the last-named author, it is our present object to unfold; and, as they are in a great measure referable to the circumstances of his professional life during the last twelve or fourteen years, a brief notice of these will be useful as well as interesting.

M. Louis, from the age of 17 to 33, studied and practised medicine in Russia with considerable success. During an accidental visit to Paris, he became acquainted with M. Broussais, whose clinique he diligently followed for some time, and who, by the novelty of his speculations, and by his zealous prosecution of pathological researches, seems to have produced a strong impression on the mind of his associate. At first he was a proselyte to his preceptor's peculiar doctrines—of inflammation being the essential or active cause of all morbid changes; and of glandular diseases being generally, if not always, consecutive to and induced by chronic phlegmasia of some adjoining mucous membrane. Experience soon taught him, however, that these, like all other general doctrines in medicine, are only partially true; and, as the maxim which he had laid down for his future studies, was to yield his assent only to what he saw and felt, he found it necessary first to question, and then to impugn the exclusiveness of the Broussaian school. Admitting that the author of the Examen des Doctrines Medicales had done much good to practical medicine, by the clear exposition which he had given of those latent forms of inflammation which had been previously so imperfectly known, and, therefore, so unskilfully treated, M. Louis was soon taught that there are other sorts or forms of morbid action, and of morbid lesions, besides inflammation, and the structural changes induced by it. He determined now to consult the book of nature with his own eyes; and, with a mind free from all prejudice and prepossession, to examine and record its phenomena for himself, and to draw his own inferences, and to abide by his own deductions. With this view, he abandoned all private practice, and entered the hospital of La Charité as a clinical clerk to his friend, Professor Chomel. For seven years, says Dr. Cowan, including the flower of his bodily and mental powers (from the age of 33 to 40), he consecrated the whole of his time and talents to minute and impartial observation. He regularly spent from three to four, and sometimes five hours a day at the hospital, devoting at least two hours to each post-mortem examination. This practice he rigorously adhered to during the whole time, being satisfied that, to observe *well*, he must not observe *hastily* ; and that the only means to rectify inevitable errors, is continually to re-examine an object, however familiar, and as if it was presented for the first time. The vast number of minutely exact observations which M. Louis was thus enabled to make, have afforded the materials for the different works which he has recently published, and among the rest, of that now before us. It is based on the results of 123 cases, the history and symptoms of which, during life, were most carefully recorded, and of which the morbid appearances found on dissection, not only in the lungs themselves, but in every other viscus and organ of the body, were regularly noted down. The immense importance of researches carried on in such a patient philosophical manner, will be better appreciated by our readers, when they become acquainted with the contents of the volume; at present we shall allude only to two or three of the most striking results, the truth of which M. Louis has the merit either of having first announced and proved, or, if they had been discovered before, of having finally and irrevocably established. The existence of tubercles in any organ or tissue of the body, after the age of 15, indicates their simultaneous presence in the lungs ; tubercles, when co-existent in the lungs and in other parts of the body, are invariably most advanced in the former—whereas, in the latter, the degree of their development is usually equal. Tuberculous deposition almost invariably commences in the upper lobes of the lungs ; it is found more frequently on the left than on the right side : simple bronchitis commences at the base of the lungs, pursuing a course inverse to that of phthisis: chronic peritonitis generally indicates pulmonary tubercles : large vomicæ are generally nearer the posterior than the anterior surface of the lobes of the lungs. These and other results of Louis' researches speedily attracted the attention of his cotemporaries ; and, although they were contradicted and cavilled at on their first announcement, their truth and value have been since very generally admitted, and have tended to establish the reputation of the author as one of the most eminent pathologists and physicians of modern times.

For the last five years he has been physician to the Hospital of La Pitié, and we are informed by Dr. Cowan, that by far the greater number of the advanced students in Paris (principally English, American, and German) have of late deserted the clinique of M. Broussais, and now follow the visits and lectures of his pupil. The distinguishing excellency of the system of instruction pursued by M. Louis is the minute and comprehensive accuracy which he practises in examining and describing every particular of a disease; all the symptoms with which it is accompanied during life; and all the phenomena which are revealed by the dissection of the fatal cases. He does not limit his enquiries to one function, or to one set of functions, although it may seem, from the prominence of certain symptoms, to be more particularly affected; he interrogates all; and the same method he follows in conducting the post-mortem examinations. Not a viscus, or part of a viscus, escapes his minute observation, and a careful record is kept of the state, whether abnormal or not, of each; for he is well aware that negative information is sometimes, especially in medical science, almost as valuable, as that which is directly affirmative.

It is proper to remark at the threshold of our enquiries, that in the following observations we limit the term "phthisis" to denote the genuine tubercular disorganization of the lungs. The symptoms of consumption may indeed be induced by other morbid changes of these organs, but certainly in a vast majority of the cases of this most fatal malady, the assertion of M. Laennec is strictly true, that the "existence of tubercles in the lungs is the *cause*, and constitutes the special character of phthisis." The division of the disease, suggested by Bayle, into the tuberculous, granulated, cancerous, melanotic, calculous, and ulcerous species, is, to say the least of the proposal, quite unnecessary. Some of these forms are of very rare, if not of very doubtful occurrence; and even when the organic lesion designated by the epithets have been discovered on dissection, they have been found very generally to be coexistent and associated with tuberculous depositions.

The terms "dyspeptic phthisis," "hepatic phthisis," and such like, involve a most erroneous doctrine. No malady of the stomach or liver can give rise to the symptoms of genuine phthisis. Phthisis may be preceded and accompanied by dyspepsia or hepatic derangements, and these diseases may predispose to a tubercular cachexia of the system, and thus may be said to induce pulmonary consumption; but to talk of the dyspeptic forms of consumption, &c. is logically improper and practically mischievous.

M. Broussais, and perhaps the greater number of the older physicians, have contended that genuine pulmonary phthisis is not unfrequently the consequence of simple chronic inflammation and suppuration of the bronchi or of the texture of the lungs, and that the existence of tubercles is by no means a necessary and essential adjunct. The great improvements which have within the last twenty years been introduced into the diagnostic department of practical medicine, independently of the more obvious features (obvious at least, and readily recognised by the experienced physician) of what we may call pseudo phthisis, are now almost universally acknowledged to afford sufficient data, to enable him to distinguish the tuberculous from any other admitted form of the disease. When we call to mind the perplexing uncertainty which so often hung over the diagnosis, and consequently the prognosis in cases of suspected consumption, before the time of M. Laennec, we cannot too highly estimate the value of his admirable labours. True it may be that he has not taught us how to cure the disease, when once it is fairly established. But this he has done: he has shewn us how to recognise its existence in its earlier periods, when judicious remedial means may do much in preventing its increase and exacerbation; and by pointing out the almost demonstrative signs of the disease in its confirmed stage, he has taught us the almost equally important lesson, of knowing when we should not do too much, and when our efforts must be limited to the soothing of the mind of our patient, and the mitigating of those bodily sufferings which are inevitable, and beyond all hopes of cure.

It is not our intention at present to enter upon the subject of phthisis in all its bearings, and to describe its causes, symptoms, pathology and treatment. This would be far too extensive an enquiry; and we shall therefore select only a few of its more interesting branches.

The researches of M. Louis having been directed more especially to the morbid anatomy of the disease, we propose, in the first place, to give an analytic review of these; then to consider the very curious subject of perforation of the lungs connected with tubercular deposition; and afterwards direct our readers' attention to some of the more important forms which consumption is apt to assume in different constitutions, and under different circumstances, describing the more prominent characters of each during life, and the more remarkable appearances found on dissection.

To those who may wish to be made acquainted with a succinct history of the disease, we most strongly recommend an attentive perusal of Dr. Clark's excellent treatise.

They will find in it an admirable resumé of all the more recent investigations and discoveries; and it is every where pervaded with the soundest practical wisdom. It is the work of a physician who has studied the disease attentively at the bed-side of his patient, with a mind unseduced by vague theory, or partiality to an exclusive system, but nevertheless richly stored with whatever is valuable and good in the writings of those who have preceded him.

### Morbid Anatomy of Phthisis.

*Lungs.*—The genuine pulmonary tubercles are almost always found associated with another morbid formation, which is known to the readers of Laennec by the name of "miliary granulations." These granulations are small, inorganized, homogeneous, shining bodies, of marked consistence, of a greyish or ash colour, more or less rounded, and varying in size from a pea to that of a millet seed." M. Louis has called them "granulations grises demi-transparentes." That they are the incipient forms of genuine tubercles, is made very probable from the following considerations:—At a certain period of their development, they exhibit a yellow opaque point at the centre, and this feature is more conspicuous in those which are found near the summits, than towards the bases of the lungs. In examining the lungs from below upwards they were generally found in the following order. 1st. Grey semi-transparent granulations;—2dly, Granulations more opaque, and of a yellowish colour towards the centre;—and 3dly, Granulations of a yellowish white in their whole extent, that is, completely tuberculous. As in the case of the genuine tubercles, the granulations are always more numerous and of a larger size in the upper than in the middle and lower lobes. These two morbid depositions are, as we have already stated, almost invariably found together in the same lung. M. Louis has in his very extended and minutely exact experience met with two cases only, in which tubercles existed, without any of the grey semi-transparent granulations; and not more than five cases in which these granulations were not associated with genuine and fully formed tubercles. It seems, therefore, extremely probable, that the grey granulations are the primary forms of tubercles, and that they become converted into these during the progress of the disease.

The granulations are sometimes scattered singly throughout the pulmonary tissue; at other times, they are clustered together in masses of greater or less extent. A case is detailed, in which these masses were of two and three inches in volume. In the middle of these masses, a number of miliary points having a character, more or less distinctly tuberculous, are usually observed; another fact, which tends to prove the connexion between these two forms of organic lesion.

The grey matter has been found in other organs besides the lungs; but it must be confessed, that this is of rare occurrence. Louis has met with two examples of it only. In the following case, he discovered it in the substance of the omentum and mesocolon. The patient, a man 27 years of age, of weak constitution, died, before the phthisical symptoms were fully confirmed. The cough was generally very feeble; the expectoration never abundant, nor distinctly purulent. Auscultation could not detect any trace of pectoriloquy or of metallic tinkling. The abdomen was more or less voluminous, with occasional uneasiness. No colic pains at any time were felt, but a troublesome diarrhœa frequently recurred.

The emaciation was very rapid; but to the last day the patient perambulated the wards of the hospital. On rising to the night-stool, he fell back and expired. On dissection, both lungs exhibited masses of grey tuberculous matter, larger and more numerous in the upper than in the lower lobes. There was no excavation, and the surrounding parenchyma was healthy. The omentum "formed a mass from twelve to fifteen lines thick, uneven, alternately yellow and blueish in colour, composed of the tuberculous and grey semi-transparent matter. The former occupied four-fifths of the mass, and was nowhere softened. The meso-colon and meso-rectum presented the same alteration, but were only half as thick as the omentum. The greater number of the mesenteric glands were tuberculous."

M. Louis appends the following remark:—

"This observation is interesting on several accounts. With regard to the tuberculous matter, it presents a solitary example, in our own experience, of its equal development in the lungs, mesentery, omentum, &c. &c., while, in our other observations, it was always further advanced in the lungs than any where else. The thoracic and abdominal symptoms were in harmony with the morbid condition of the organs.

The re-union of a certain quantity of the grey semi-transparent matter with the tubercular in the omentum, is an additional argument in favour of their mutual connection." 100.

But while we contend for the very intimate relation between the grey semi-transparent deposit and the genuine tuberculous formation in the case of the lungs, we must admit that even in them, the latter of these two changes may sometimes be developed primarily and without the antecedence of the grey granulations.

"The identity of the grey semi-transparent granulations with tubercle, has been a source of much discussion. Bayle thought them cartilaginous—Andral (*Clin. Med.* vol. iii. p. 5,) indurated pulmonary vesicles—Chomel (*Dic. de Med.* vol. x. Article 'Granulation') says, they are *not* tubercles, but withholds his reasons. Bouillaud seems to agree with Andral, Lombard entertains a very similar opinion, &c. We only mention these hypotheses to attract the reader's attention to the evidence adduced by our author; it is we think conclusive, and coupled with other facts distributed throughout this volume, demonstrates some *neecssary* relation between these two alterations, as strongly, as it is in the power of facts to witness in favour of two things which are not *absolutely* identical. (Translator.)"

We have already alluded to the important fact of the upper lobes of the lungs being almost always affected with tuberculous disease, in preference to the lower. Indeed the very apex of the upper lobes is the part which is usually most early and most extensively tuberculous.

Large excavations are almost exclusively found in the upper lobes; and it is of very rare occurrence, that even a small vomica is discovered in the lower lobes, when the upper ones are altogether exempt.

Another important feature of tuberculous deposition is, that it appears to take place more frequently on the left, than on the right side. We admit that in a large majority of cases, both lungs are simultaneously diseased. M. Louis has however met with a few examples of tubercles having been limited to one side; five times to the left lung, and twice only to the right. He has also found excavations to be more frequent on the left than on the right side; out of eight cases of vomicæ which had opened into the cavity of the pleura, seven were observed on the left side. This opinion, as to the greater frequency of tuberculous deposits in the left lung, is contrary to the experience of M. Laennec (vide Forbes' Translation, p. 282), but in unison with that of Dr. Stark (Medical Communications), and also of Dr. C. Smyth, who deduces his opinion from an examination of the cases in the works of Bonetus, Morgagni and others.

With respect to the portion of the lobe, or lobes, affected with tuberculous deposits, the following observations of our author deserve to be generally known.

"The great tuberculous excavations of the upper lobe were nearer the *posterior* than the anterior edge of the lung, and in many instances we have found their sides in the former direction, almost wholly formed by a false semi-cartilaginous membrane, from a line to a line and a half in thickness, enveloping the summit of the organ. Inferiorly, they were sometimes only separated from the pleura which covers the interlobular fissure, by a thin layer of pulmonary tissue, more or less modified—(*Obs.* 28),—or there was a perforation of their parietes in this point, and communication established between another excavation, situated in the inferior lobe and posteriorly; for it is worthy of remark, that in no one instance have we met extensive excavations in the centre of the lower lobe." 10.

Before leaving the subject of tuberculous lesions of the lungs, it is necessary to allude to an opinion maintained by M. L. as the result of his extensive pathological enquiries, which is opposed to the doctrines of Laennec,

Andral, and some other distinguished physicians. Our author says " in no one instance have we met, surrounded by *healthy* pulmonary parenchyma, with cavities communicating with the bronchi, and lined, as are tuberculous excavations of long standing, with a false membrane of a light grey colour, semi-cartilaginous and semi-opaque ;" and he adds, " we have also failed to meet with those masses of condensed cellular tissue, in which the bronchial ramifications, more or less dilated, terminate, and which are considered by Laennec as the cicatrices of tuberculous cavities."*

He is however too candid and enlightened an observer to call in question the authenticity of the observations made by such an anatomist as M. Laennec, who has recorded several instances of vomicæ having been found to be surrounded by healthy pulmonary parenchyma in the bodies of patients who had presented the symptoms of phthisis during a space of time more or less considerable; and "judging from the structure of these cavities it would be difficult not to believe that tuberculous softening preceded their formation."

In the work of Laennec, Andral, Cottereau (Essay on Chlorine Inhalation) our readers will find numerous cases adduced to prove the cicatrization of tuberculous excavations, and the formation of a fibro-cartilaginous membrane, when the cavity has not been obliterated. We have however no doubt, but that many cases of cicatrised vomicæ, which have of late years been recorded, are of very doubtful authenticity. The error of mistaking every puckered depression on the surface of the upper part of the lungs, has been, we fear, very often committed, even by some of the best authorities on pathological anatomy. These depressions do not always depend upon any determinate lesion. They were frequently observed, when the texture of the lungs was healthy, or only slightly indurated to a small depth, immediately beneath the pleura. In other cases they were found associated with crude tubercles, small excavations, or osseous concretions in the summit of the lungs.

*Pleuræ.*—No other morbid phenomena (not including the tubercles themselves) are of such frequent occurrence in dissections of phthisical patients, as adhesions of greater or less extent, between the surfaces of the pleuræ. In 112 cases, there was only one in which both lungs were free in the whole

---

* " These results are singularly negative, when compared with those of M. Laennec, Andral, and others, who bring forward *copious* and *undeniable* evidence of cicatrisation of tuberculous excavations, and the formation of a fibro-cartilaginous membrane, when the cavity is not obliterared. It must be recollected that M. Louis never forces his conclusions beyond the number of facts he is analysing, and it is remarkable that not one of these has presented an example of cicatrisation ; this inclines us to suppose that the presence of a cicatrix has often been hastily admitted ; a supposition confirmed by the succeeding observations of our author. That a tuberculous excavation is *ever* capable of cure is an important fact, and highly calculated to encourage us in the research of means calculated to arrest this hitherto most destructive affection. Vide Andral, *Clin. Med.* vol. iii p. 382 ; Laennec, pp. 299, 323—and notes by Dr. Forbes. Cottereau, in his *Essay on Chlorine Inhalation,* adduces also some incontestable facts. (Translator.)"

of their extent: in eight cases the right lung, and in seven, the left one, did not adhere at any point of their surfaces. In all of these cases there were either no tuberculous excavations, or if there were, they were only of very small dimensions. The extent and firmness of the adhesions were very generally proportionate to the number and size of the tuberculous excavations in the lungs. The observations of M. Louis on this subject cannot be advantageously abridged.

"There was evidently some relation between the extent of organic alteration and the pleural adhesions; if the latter were absent, there were neither large nor middle-sized excavations, and in general, none whatever. Were they weak and limited in extent, the cavities were generally very small, and sometimes altogether wanting.

Lastly, where the adhesions were dense, extensively distributed or even universal, they *always* indicated excavations in the lungs, and in the great majority of cases, that those excavations were large, or at least of considerable size.

The proportion which existed between the size of the tuberculous cavities and the adhesions, demonstrated the influence of the first upon the second. The large excavations constantly occupied the summit of the lungs, approximated closely to their surface, and there only, were found those dense resisting false membranes, which we have already described as either strengthening the sides of the cavity or constituting them entirely. This mutual relation between cavities and adhesions is also pointed out by other facts. Thus, in two cases where the lungs presented only two masses of tuberculous matter immediately beneath the pleura, the adhesions were confined to these points, and were formed by cellular prolongations of corresponding extent." 29.

A pathological phenomenon of much interest, but of very rare occurrence, is the deposition of genuine tuberculous matter in the false membranes, between the surfaces of the pleuræ. Under such circumstances, the false membranes are found not unfrequently to have acquired quite a cartilaginous firmness. They are usually observed, enveloping the summit of one, or of both lungs. From the frequency of pleuritic attacks in the last stage of pulmonary phthisis, (M. Louis observed it in one tenth of his cases): a very common appearance on dissection is a layer of soft yellowish coagulable lymph at some point, or points of the pleura, which has been evidently of very recent deposition; within from 4 to 18 or 20 days preceding death.

An effusion into the cavity of the pleura of a clear fluid, in quantity from a pint and upwards, was found in one tenth of all the cases. The effusion had in most instances taken place very rapidly. "Of this," says M. Louis, "we were convinced in two instances, where the thorax gave every where a clear sound on percussion thirty-six hours before death, but where two pints of fluid were afterwards found in one side of the chest."

*Trachea.*—The most frequent lesion of the trachea in phthisis, is ulceration of its mucous membrane, with, or without an inflammatory redness of its surface. It was observed in 31 out of 102 cases examined. When the ulcers are small, they are usually scattered throughout the circumference of the air-tube, are of a round or oval shape, and vary from a line to a little more or less in diameter. From their edges being flat, and their bases being formed by the cellular tissue slightly, or not at all thickened, they often appear as if they had been artificially produced; and hence they have escaped the attention of many pathologists.

The ulcerations are usually more numerous, and of a larger size in the lower, than in the upper half of the trachea.

The large ulcerations are more scattered and apart from each other than the smaller ones, and they are very generally found on the posterior fleshy part of the tube.

" A certain number of the cartilaginous rings were sometimes completely denuded, diminished in thickness, and either partially or *wholly* destroyed. This last alteration we have only observed twice ; while we have seen in five cases the complete destruction of the mucous membrane of the trachea, throughout almost the whole extent of its fleshy portion." 32.

The symptoms of ulcerated trachea are very obscure. Pain, accompanied with a sense of burning, and of an obstruction, just above and behind the sternum, was experienced in a few of the cases. Sometimes the pain and uneasiness were referred by the patient to the larynx, although this part was exempt from the disease, and the trachea only was ulcerated; just as we observe that the pain in cystitis is very often felt in the extremity of the urethra, and not in the seat of the disease.

The distress in the breathing, the cough, and the expectoration presented no peculiar, or pathognomonic characters. In one case in which the whole length of the windpipe, from the epiglottis, including this appendage, to the termination of the trachea, exhibited patches of deep and extensive ulceration, so that even some of the cartilaginous rings were denuded, and others were more or less completely destroyed, no prominent symptoms were present during the life of the patient.

Simple inflammation of the mucous membrane of the trachea is more frequently accompanied with pain and a sense of heat in the affected part, than ulceration is.

*Larynx.*—The ulcerations of this portion of the windpipe are less frequent than, but are very rarely unaccompanied by, those of the trachea. They were found in 22 out of 102 cases.

" Seldom superficial or presenting the appearance of artificial formation, they were generally of a certain depth, more or less irregular, and from one to two lines broad. Their edges, of variable consistence, were sometimes lardaceous, of a greyish or whitish colour. The mucous membrane was pale and perfectly sound in the rest of its extent.

The most frequent seat of these ulcerations was, first the junction of the vocal cords, where they were sometimes superficial; then the vocal cords themselves, especially their posterior part; we have only once observed a very small ulceration at the base of the arytænoid cartilages, the superior part of the larynx, and the interior of the ventricles.

In some instances, one or more of the vocal cords were completely destroyed, and the base of the arytænoid cartilages was laid bare. When this was the case, the cartilages themselves were unaffected." 34.

The signs, during life, of ulcerated larynx are much more obvious and uniform than those which accompany ulceration of the trachea. They vary according to the part of the tube affected, and also according to the depth and extent of the ulcers. When the chordæ vocales, the ventricles, or the arytænoid cartilages were the seat of disease, hoarseness, with more or less alteration of the voice, pain, sense of heat and pricking, and subsequent aphonia, were generally present. The pain was sometimes very acute, pungent, and lancinating, and, in most of the cases, it was exasperated by coughing, speaking, &c.

*Epiglottis.* The morbid appearances of this appendage have hitherto been overlooked in the examination of phthisical bodies, although ulcerations of its surface are almost as frequent as are those of the larynx. They were seen in 18 out of 102 cases. The cause of this omission, M. Louis rightly ascribes to the circumstance of anatomists seldom examining attentively any organs, except those whose functions are obviously disturbed during the life of the patient. Although occasionally found alone, the ulcerations of the epiglottis are, in most cases, associated with similar lesions of the larynx and of the trachea; out of eighteen observations, in which ulcerations of the epiglottis were found, in six only were the larynx and trachea exempt from them. They are almost always confined to the lower surface of the valve—once only has M. Louis found them on its upper or lingual surface.

"In some cases the mucous membrane of the epiglottis was destroyed over the whole extent of the inferior surface. In others, the cartilage was destroyed in portions of its circumference, giving a festooned appearance to the epiglottis. This we have seen four times. A fifth case has presented an example of complete destruction of the epiglottis.

We have in no one instance discovered tuberculous granulations in the substance, or on the surface of the epiglottis, larynx, or trachea; inducing us to believe that we ought to consider inflammation as the most frequent cause of the ulcerations.

Another fact of importance to remark is, that these ulcerations were twice as frequent in men as in women. Thus, in an equal number of cases, the women only presented six examples of this state of the epiglottis, seven of the larynx, and nine of the trachea, out of eighteen, twenty-three, and thirty-one cases; and as the proportion is nearly equal for the three kinds of ulcerations, it is probably not the effect of hazard." 35.

The symptoms of ulceration of the epiglottis, although often obscure and unsatisfactory, may be stated to be, a fixed pain in the upper portion of, or immediately above, the thyroid cartilage, soreness of the throat, hoarseness, and a greater or less degree of dysphagia, and which increases so much during the progress of the disease, that in the effort of swallowing, fluids are perhaps rejected by the nostrils, the pharynx and tonsils remaining healthy all the time.

In one case, in which the epiglottis, the lateral ligaments, and the superior vocal cords were found completely destroyed, the succession of the symptoms was well marked. At first, the voice was hoarse, unequal, and discordant; a lancinating pain was felt between the thyroid cartilage and the os hyoides; this was increased by any exertion of the vocal organs, by flexion of the neck forwards, and by deglutition; the latter was frequently difficult, and provoked the rejection of fluids by the nose. Towards the close of the case, the pain in the neck became more severe, and the deglutition so distressed that no solid food could be swallowed: complete aphonia had supervened. The progress of the symptoms in this case had been slow and constant—the rejection of drinks by the nostrils had existed for four months preceding the death of the patient; and the acute local pain had been felt during the whole of this period. It is, however, to be remembered, that these symptoms are by no means of invariable occurrence, even when the destruction of the epiglottis is complete. Majendie relates two such cases, where deglutition was not at all impeded.

Having thus treated, at considerable length, of those structural lesions of the respiratory organs which have been observed by M. Louis in the bodies of those who have died of genuine phthisis, we proceed to lay before our readers an analysis of his researches on the morbid changes in the other organs of the body; and first with respect to those in the Circulating System.

*Heart and Pericardium.*—The opinion which has been held by some physicians, that phthisis is one of the causes of aneurism of the heart, is contradicted by the experience of M. Louis. In 112 cases, there were only three in which he detected any increase in the size of the heart. This increase was confined to the left ventricle, and none of the patients had experienced, during life, the symptoms of hypertrophe, or of dilatation of the heart. "In the great majority of cases, the heart was under its usual dimensions, being not more than one-half or two-thirds of its natural volume." Dr. Clark has stated, in his work on Climate, that he considers a small, feeble heart to be a strongly predisposing cause of consumption; whereas M. Broussais says that he has observed hypertrophy of the heart to be sometimes the cause of phthisis, and yet that it may become atrophied, in the progress of the disease, with the other organs.

The latter clause only of M. Broussais' observation is confirmed by the experience of M. Louis, who distinctly asserts that, "all that we can possibly conclude respecting the influence of phthisis on the heart is, that its volume is diminished in common with that of the other organs." The truth of this remark is amply confirmed by the fact, that the heart is very often found to be diminished in volume after other chronic diseases, as well as after phthisis. M. Louis found it smaller than natural in 30 out of 80 such cases; a higher proportion than in phthisis. After organic affections of the stomach and uterus, this diminution was more frequent, and more strongly marked, than after any other diseases.

Effusion of serum into the cavity of the pericardium was found in one-tenth of the cases of phthisis examined by M. Louis.

The morbid appearances observed in the aorta are not sufficiently frequent in their occurrence, nor uniform enough in their characters, to be entitled to much consideration in the pathological history of pulmonary phthisis. The internal surface is sometimes of a deeper red than usual; and, at other times, we meet with various organic changes, which are, however, quite as common after other chronic diseases as after phthisis.

The organs which next claim our attention, are those which belong to the Digestive System.

The state of the *pharynx* and *œsophagus* need not detain us a moment, as "they were almost constantly healthy." The *stomach* much more frequently presents some morbid phenomena. M. Louis believes that this organ is enlarged in volume more frequently after phthisis than after any other chronic disease, and he is inclined to regard this change "as the consequence of repeated shocks caused by the cough." But surely this is rather a fanciful opinion; and his translator has, with much propriety, expressed his dissent in a foot-note, argueing that the explanation offered by

M. L. is improbable, "both from the physical laws which regulate the abdominal cavity, and the fact that in phthisis the cough is not so violent, or so protracted, as that accompanying some other thoracic affections." The lesions of texture usually found in the stomach after phthisis are certain alterations of its mucous membrane.

"This was both thinned and softened—sometimes even destroyed; in other cases it was more or less red, and occasionally thickened in its anterior surface; while again in others the redness, accompanied by considerable softening, existed only in the left extremity. Ulcerations were sometimes found; but more frequently there was a remarkable *mamillated* appearance of the mucous membrane." 44.

Our limits forbid us from entering upon the minutely detailed descriptions of these several lesions, given by M. Louis. The pathologist will be amply rewarded by diligently perusing them at his leisure. We can afford room for one short extract only. "The stomach was more or less morbidly affected in four-fifths of the cases of phthisis, and in not more than one-half of the cases of other chronic diseases. It may also be noticed, that while in phthisical patients the most considerable morbid change, (viz. softening, with diminished consistence, and sometimes destruction of the mucous membrane), was one of the most frequent, the contrary was the case in those who died from other diseases."

The pathology of the *small intestines* in phthisis offers a much more extensive and more important theme of reflection than that of the stomach, from the great frequency and gravity of the morbid changes which they present. Of these changes, by far the most common is ulceration affecting the mucous follicles, and the surrounding mucous and cellular textures. Louis has detected it in five-sixths of the cases which he has examined; a proportion which is higher than what is admitted by MM. Bayle and Andral, but which he thinks will be found to be strictly correct by succeeding enquirers, if they will employ sufficient time and attention in properly cleansing the small intestines, (the jejunum and ileum: the duodenum is very rarely the seat of this, or of any other lesion), and in scrupulously examining them throughout their whole extent. The portion of the gut most frequently affected with these ulcerations is the lower half or two-thirds of the ileum. "When small they are almost exclusively situated opposite to the mesentery, in points corresponding to the aggregated glands (glandulæ Peyeri) which were themselves destroyed. In their maximum of development, they occupied the whole circumference of the intestine." The shape of these ulcerations is generally found to vary according to their size: they are rounded, when small; and more elliptical, corresponding to the shape of the clustered glands, when of larger dimensions. They are sometimes, although very rarely, lineary.

"The large ulcerations were frequently the result of the junction of smaller ones; a fact easily demonstrated when the latter were numerous and situated among the agminated glands. There might then be observed softened tubercles, with small circular ulcerations, separated by entire or partially destroyed bands. In others, no remnant of these divisions remained, the cellular membrane was completely denuded, more or less thickened, and presenting small round shaped depressions of variable depth, corresponding no doubt to the partial ulcerations just described. Lastly, in a third division of similarly formed ulcerations, the sub-mucous layer was either in part or wholly destroyed, and the muscular coat denuded, uneven, and thickened." 62.

These ulcerations are usually preceded by enlargement and induration of the two sets of glands, the solitary and the aggregate, which are distributed along the inner surfaces of the small intestines. M. Louis applies the term "granulations" to these enlarged mucous glands. He describes two sorts of them; one presenting all the characters of tuberculous matter, the other being harder, whiter, and having a cartilaginous aspect. Their size varies from that of a small shot, or even of a pin's head, to that of a common pea. They are almost invariably accompanied by ulceration. The semi-cartilaginous granulations are in general much more numerous than the others; they were sometimes seated among the aggregated glands, but more frequently in the intervals between the different clusters. M. Louis observes that they are situated immediately beneath the mucous membrane, and never among the fibres of the muscular tunic. When these granulations become ulcerated, the edges of the ulcers are indurated, white, and opaque, "retaining," says M. L., "almost exactly the characters of the tumor to which they succeeded: thus pointing out the nature of their cause." The other sort of intestinal granulations, the tuberculous, differs in some important respects from the cartilaginous sort. They are admirably described in the following paragraphs :—

"These were also situated either round the ulcerations, in their centre, or in the interstices of the muscular fibres; between these and the peritoneum;— among the agminated glands or in their intervals; and were almost constantly more numerous near the cœcum than elsewhere. We have never found them near the duodenum.

These granulations were succeeded by small ulcerations, produced by the same process as are tuberculous excavations of the lungs. The tuberculous matter gradually softened, and the mucous membrane was proportionably red, thickened, and softened in the corresponding point; if destroyed, the contents of the abscess were emptied on the intestinal surface, so that inflammation of the mucous membrane was here an effect and not a cause of tubercles.

We have never seen tuberculous matter occupying the intestinal mucous membrane under any other form than that of granulations." 59.

In concluding our remarks on the subject of intestinal granulations in phthisis, it is worthy of notice that they are less frequently observed than the ulcerations described in the preceding page. They existed thirty-six times in ninety-five cases, whereas the ulcerations were found seventy-eight times in the same number. Whether the ulcerations are ever quite independent of any previous enlargement of the mucous follicles, has hitherto not been satisfactorily determined.

The other lesions of the small intestines, which are found after phthisis, such as softening of the mucous membrane, thickening of this membrane, the occurrence of small tuberculous abscesses, (independent either of granulations, or ulcerations,) are of minor importance, and need not detain us. All of them, and indeed we may add to the number, the semi-cartilaginous granulations, are found (but certainly not so frequently) after other chronic diseases, as well as in phthisical cases; whereas the tuberculous granulations and ulcerations are peculiar to phthisis.

"We have never," says M. Louis "observed these last-named lesions, except in phthisis; and if it is not rigorously correct to say that ulcerations of the small intestine are exclusively found in this affection, exceptions are so rare, that the proposition is almost literally true."*

Dr. Cowan informs us that, during the eight years which have elapsed since the publication in France of the present work, M. Louis has not examined a single subject who died from a chronic disease, and presented ulcerations in the small intestine, in which he did not find co-existent tubercles in the lungs.

*Large Intestine.*—The lesions of this portion of the alimentary canal are similar, with the exception of the cartilaginous granulations being never found, to those which we have described, as occurring in the small intestines. The mucous coat was sometimes found unusually red, the redness being either in patches, or for a continuous surface of considerable extent. This appearance almost always coincided with marked softening, and very frequently at the same time with a thickened state of the membrane, a condition, which, it can be scarcely doubted, has an inflammatory origin.

The red and softened mucous membrane presented very often a mamillated appearance; sometimes innumerable minute abrasions; and in other cases, a continuous surface of many inches similarly abraded; an appearance which might have readily escaped notice, in consequence of the submucous tissue having a slightly faint tinge, and thus resembling the healthy mucous surface. In one eighth of all the cases examined, tuberculous granulations were found in the large intestines. These were situated either in the centre, or round the circumference of the ulcerations, and seldom or never in their intervals. Ulcerations were nearly as common in the large, as in the small intestines. Usually they were of a small size—from three to six lines, or thereabouts, in diameter, rounded, and, with flattened edges, so that they looked as if they had been artificially produced. In a few cases, the whole length of the large intestine, presented an infinitude of minute ulcerated points, so indistinct that they might be readily overlooked by an ordinary observer. When the ulcerations were numerous, and the intervening mucous membrane was more or less thickened, the general aspect very much resembled the chapped integuments of the hand. The larger ulcerations were of a more irregular shape, and their edges were dentated, or radiated. Sometimes they were of so large a size that not only the whole circumference of the gut at one or more points, but even a longitudinal space of from six to nine inches in extent was occupied with these ulcerations.

The largest were usually observed in the cœcal and in the ascending portions of the gut. In the rectum, they were never found larger than from an inch and a half to two inches in length. The relative frequency of ulcerations (including the small and the large) in the different portions of the large intestine, viz. the cœcum, ascending, transverse, descending colon and rectum, may be stated by the following figures: 34 : 37 : 25 : 8 : 32:—that is, in the same proportion of cases, they are almost equally common in the cœcum and rectum. The concluding observations of our author are not

* It is to be observed that M. Louis's remark is applicable only to chronic diseases. He does not include typhus fever.

only so practically valuable, but afford, at the same time, so just a commentary on the leading doctrines of the Broussaian school, that we must find room for them, although the extract is a long one.

"Intestinal ulcerations were often, at least in their origin, independent of inflammation. This was evidently the case with a great number of those in the small intestine, the result of softened tubercles ; for the development of the latter could not be attributed to inflammation, since so long as they remained unsoftened, the mucous membrane covering them, continued healthy. Far from being the cause, the inflammation of the mucous membrane was, as we have already seen, subsequent to the presence of the granulations. The same remark is equally applicable to some cases of ulceration of the large intestine.

Where softened tubercle could not be considered the cause, it would still be difficult to regard ulceration as simply the effect of inflammation, which does not usually take place in isolated patches on a mucous surface. Of this fact we have, we think, afforded an example, when speaking of the softening with redness and thickening of the mucous membrane of the colon, which almost invariably extends to its whole surface. As to the small intestine, we will remark, that while distinct traces of inflammation are much less common than in the colon, its ulcerations are still more frequent; and that where inflammation appeared to be the cause, it had still a peculiar character, being most generally bounded to the portion of mucous membrane occupied by the agminated glands.

These reflections are strengthened by what we have said respecting the extreme rareness of the ulcerations of the small intestine in all chronic diseases except phthisis ; while simple inflammation of the mucous membrane is quite as frequently observed in one case as the other." 72.

*Lymphatic Glands.*—Tuberculous deposition in some of the clusters of these glands is of frequent occurrence after phthisis, (having been found in a fourth of all the cases examined by M. Louis) and is according to his researches indicative of, or peculiar to, at least in patients of 15 years and upwards, this disease.*

In several hundred dissections of cases, which have proved fatal from other chronic maladies, (and in many of these, the intestinal mucous membrane was extensively inflamed and ulcerated,) our author has not met with a single instance of tuberculized lymphatic gland.

Whenever, therefore, he detected this lesion, he assures us, that his prediction of the lungs being similarly affected, was invariably verified by the post-mortem examination. That the morbid change in the glands is very generally preceded by a corresponding change in the lungs, is made probable by the circumstance of his always finding that the tuberculous matter was more advanced in the lungs than elsewhere, and that when tubercles co-existed at the same time in different parts of the body, these were almost constantly at the same degree of development.

The mesenteric lymphatic glands are more frequently tuberculized than any other groupe. M. Louis found them diseased 23 times in 102 cases.

* From the enquiries of M. M. Andral and Lombard, it is made evident that tubercular disease of the lymphatic glands, including the external, as well as the internal groupes, is not only much more common in infants and young children under 12 or 15 years of age, than after that period of life, but also, that it is more frequently independent of, and unassociated with the existence of pulmonary tubercles, in the former, than in the latter class of patients.

In the majority of cases the change did not affect the whole substance of the glands, but was partial and disseminated in points through their substance, which was usually redder and softer than in health. The grey semi-transparent matter is very rarely seen in these or in any other glands, the deposition of the genuine yellow tuberculous matter appearing to take place primarily and independantly of any antecedent change. The tuberculous matter is rarely found to be in a softened condition in diseased glands. In every instance where the mesenteric glands were tuberculous, ulcerations of the intestines were found; and as these ulcerations were most common, in the lower portion of the ileum, and in the cœcal portion of the large intestine, the glands in the adjoining mesentery were oftener found diseased, than any others.

The lumbar, cervical and axillary groupes of glands were more or less tuberculous in about one-tenth of the cases examined; and, as in the case of the mesenteric glands, they were not unfrequently found merely enlarged, and of a variably intense red colour, without any positive degeneration of texture.

We might have expected that the cervical glands would have been more commonly affected, considering their proximity to, and connexion with the primary seat of the disease, than they appear from the tables of M. Louis to be. His inference from this fact is therefore probably quite correct, that "tuberculous transformation must be viewed as depending upon some other cause than the inflammation of the corresponding mucous membrane." With respect to the state of the bronchial glands in phthisis, M. Louis informs his readers that, he is not prepared, from his own personal observations, to state accurately the character and frequency of their lesions in phthisis. M. Lombard, in his Essai sur les Tubercles, has in some degree supplied this deficiency. From his tables, it appears that these glands were found diseased only nine times in 100 adult phthisical cases, and no fewer than eighty-seven times in the same number of cases occurring in infants and young children.

*Liver.*—The most frequent and most remarkable change of this viscus, in phthisis, is the fatty degeneration of its substance.

"It existed in one-third of the cases (40 out of 120.) In this condition it was pale, almost always of a light brownish yellow colour, spotted with red, externally and internally. It retained its natural form; but its volume was nearly always augmented, and at times double its usual dimensions. This increase was almost invariably at the expense of the right lobe." 79.

The texture of the liver is usually much softened, so that it may be readily torn and broken down by the fingers. When the morbid change is recent and incomplete, its existence may be most easily shewn by placing a thin section of the liver on a sheet of paper, and exposing it to a gentle heat; the fatty matter is melted, and saturates the paper. In the more advanced stages, the scalpel and fingers of the anatomist are greased as by common fat. This fatty alteration of the liver is much more frequent in female than in male subjects. In forty-nine cases observed by M. Louis, ten only occurred in the latter. Age did not appear to have had any influence in predisposing to this lesion. Some pathologists have supposed that there was some connexion between the fatty change of the liver, and diseased condition of the duodenum. The researches of our author do not confirm the accuracy of this opinion. He has not been able to discover any set of symptoms which are indicative, during life, of this morbid alteration of structure; but, as the increase in the size of the liver in cases of phthisis, (a very common occurrence,) is almost always owing to, or at least co-existent with, the fatty degeneration, we may expect very generally to discover the latter, in such cases, on the dissection of the body. According to the researches of M. Louis, it is almost peculiar to pulmonary consumption, and may, therefore, be regarded as somehow or other depending on this disease.

"Out of 230 cases, nearly equally divided between acute and chronic diseases, we have only met nine examples of fatty liver; and among these nine, seven relate to patients who presented a certain number of pulmonary tubercles. By adding these nine cases to the forty already mentioned, we have forty-nine examples of this condition of the liver (and these include all we have collected during three years), out of which forty-seven were cases of phthisis. There are assuredly few phenomena of whose mutual dependence there is less doubt, and in confirmation of which facts are more unanimous." 80.

All other organic lesions of the liver are extremely rare after phthisis. M. Louis has only twice remarked the deposition of tuberculous matter in its substance. When the liver was in a state of adipose degeneration, the bile was usually found of a viscid, treacle-like consistence, and of a very dark colour. But this condition of the bile is by no means pathognomonic of this morbid change of the liver; it is observed to accompany, although more rarely, other lesions of this viscus.

*Spleen.*—Genuine tubercles were found in this organ seven times in 90 cases, examined by M. Louis. They were very numerous, varied in size from that of a hemp-seed to that of a filbert, and exhibited all the characters of genuine pulmonary tubercles. The greater frequency of tuberculous deposits in the spleen than in the liver, is deserving the notice of the pathologist.

There was another sort of deposit occasionally observed in the substance of the spleen; it consisted of "rounded, yellowish, shining, elastic, moist granulations, very different from the tubercle." The size of this viscus was occasionally larger, at other times smaller than in health. Its consistence was equally variable in different cases.

*The Kidneys* were perfectly sound in three-fourths of the cases. Three times only did they exhibit any tuberculous changes. The *bladder*, more or less contracted or distended, was never found diseased. Tuberculous matter was discovered in a very few instances, in the *prostate gland* and in the *vesiculæ seminales*, on the surface of the mucous membranes of the *ureters* and *seminal ducts*, and also in the substance of the *uterus* and of the *ovaries*. Its occurrence was occasional, but certainly very rare in any part of the *peritoneum* itself, or in the *false membranes*, which were not unfrequently found covering the *intestines* and the anterior parietes of the abdomen. In one remarkable instance, it was observed in the *omentum* and *mesocolon*.

Such is a brief summary of the more frequent and striking changes witnessed in the abdominal and pelvic viscera of patients who have died of phthisis. We shall now rapidly glance at those which have been discovered by M. Louis in the *brain* and its investing *membranes*.

None of the encephalic lesions, tubercles excepted, (and these are of very rare occurrence), are peculiar to, or indeed are more frequently observed after, phthisis than after other chronic diseases. The thickening of the arachnoid, the granulated state of this membrane near the falx, the softening of portions of the brain, and the partial effusions of serum, either between the membranes or within the cavities of the brain, are common post-mortem appearances in cases of protracted illness, and all of these lesions, we know, are compatible with the persistence of the mental powers in their full vigour. The occurrence of tubercular deposit in the substance of the brain, in adults, is so rare, that an abstract of the appearances, in the only case recorded by M. Louis, may be deemed interesting. The patient, a young girl aged 19, had suffered from violent headache, and there had been much drowsiness and occasional pervigilium.

*Dissection.* "At the posterior part of right hemisphere, the arachnoid was adherent to the dura mater, in a point corresponding to a nodulated tumour, developed near the surface of the brain, and about the size of a common nut. It was of greenish-yellow colour, firm, in every respect tuberculous, and *not encysted.* Round it the cerebral substance was healthy. Between the upper surface and lateral ventricle of same hemisphere, five similar tubercles existed. On the left side there were four, and one of them occupied the posterior and inferior part of the opticus thalamus. At the base of posterior lobe of same side, a portion of the cerebral structure was transformed into tuberculous matter, under the form of a layer four lines thick, and an inch and a half in extent. It was partially adherent to the falx cerebelli, the corresponding layer of which had undergone the same alteration. Lastly, at the inferior part of left hemisphere of cerebellum, a non-encysted tubercle, about the size of a nut, extended to the spinal marrow, intersecting a certain portion of its substance." 112.

The lungs exhibited several vomicæ, and much tuberculous infiltration, and this last-mentioned change was seen in the cervical, axillary, and mesenteric glands, and also in the spleen ; but no where, except in the lungs, had the tuberculous matter undergone any softening, or other ulterior development.

From the tenor of the preceding observations, the attentive reader will be prepared for the general conclusions respecting phthisis, which M. Louis has drawn from his extended experience. Tubercular deposit is, according to his views, the essential precursor or cause of the disease ; and this morbid process invariably commences (in patients, at least, of 15 years and upwards) in the pulmonary structure. In not a single case, out of many hundreds, has he ever found tubercles in other parts of the body, when no trace of them could be detected in the lungs. To this pathological axiom we may add two others, viz. that, in every instance, the development of the tuberculous matter in the lungs was more complete, and farther advanced, than elsewhere, as, for example, in the spleen, intestines, prostate, &c.; and that, in these viscera, however distant and dissimilar from each other, the tuberculous matter was uniformly in the same stage or degree of development—"a coincidence which seems (says M. Louis) to indicate the existence of a common cause, acting at once on all these points, and which inclines us to believe that tuberculous deposition is independent of those occasional causes (he alludes to the existence of inflammatory or congestive action, which is supposed by Broussais, Andral, and others, to be an invisible and necessary precursor of this lesion) which we are apt to suppose active in certain cases."

These conclusions of our author are, it must be remembered, at variance in some degree with those which other enquirers of great eminence have drawn from their investigations. Andral appears to have found tubercles in various organs, when none could be detected in the lungs, more frequently than M. L. is willing to admit; and he objects to the reasoning of our author, that, because the tubercles of the lungs are almost always found further advanced and developed than those in other parts of the body, it follows, as a necessary consequence, that the deposition must have taken place primarily in the lungs, and consecutively only in every other viscus or tissue. The peculiar functions of the lungs, their more frequent exposure to irritating influences, and the unceasing movement to which they are subjected, may account for the greater rapidity of development, when the deposition has been once established. M. Lombard, of Geneva, in his ingenious Essay on Tubercles, has adopted similar views to those of M. Andral. He does not admit the supposed necessary co-existence of tubercles in the lungs, even in adult subjects, whenever these bodies are detected in other parts of the body. The question, therefore, is open for future investigation, and may and ought to be examined, and, if possible, speedily decided, by those physicians who have extensive opportunities of prosecuting pathological enquiries.

How is it that the English hospital physicians are such idlers in the vineyard of science? With the exception of Dr. Bright, we can scarcely mention one who has even evinced any zeal, far less made any original contributions, to a cause, which has been so splendidly illustrated by the labours of Bayle, Laennec, Andral, Louis, and many others of the French school. Drs. Abercrombie, Carswell and Hope, (who along with Dr. Bright have been by far the most meritorious among recent British pathologists,) are, or at least were not, when they published their respective works, attached to hospitals. Would that we could animate the functionaries of St. Bartholomew's, St. Thomas's, and some other of our metropolitan institutions, from the quiet slumber of their repose. They really should bestir themselves. It is "too bad" that while our hospital surgeons have honourably distinguished themselves among the writers of the day, our physicians (even although many of them have drank of the pure stream of professional knowledge from the Isis and Cam!) are little known beyond the boundaries of Middlesex. Let them remember that our medical literature does not possess one indigenous manual of morbid anatomy, except that of Dr. Baillie, published forty years ago; that we are obliged to be contented with translations from the works of our more active Continental brethren; and that even these translations have been but seldom undertaken by the very men, who enjoy the requisite opportunities of testing the accuracy of the author's assertions, we mean hospital physicians.

We now proceed to consider the highly interesting subject of

PERFORATION OF THE LUNGS, in connection with tubercular disease of these organs. The symptoms of this accident are almost always sufficiently prominent to announce the fatal mischief that has taken place. The sudden, or even instantaneous seizure of extreme difficulty of breathing, amounting, perhaps, to dread of immediate suffocation, the acute pain at one point of the chest, the appalling anxiety, the blanched features, and the weak or imperceptible action of the heart and of the arterial pulse, all these symptoms coming on when the patient is perhaps comparatively easy and free from suffering, may be considered as sure and positive signs of the lung having given way, and having permitted the escape of the air, and it may be of some fluid also into the cavity of the pleura. In eight cases, which have occurred to M. Louis's observation, all the symptoms now enumerated were, with the exception of the pain being absent in a solitary instance, so strongly marked, as to lead him to form a correct diagnosis at the time. According to his experience auscultation and percussion may indeed confirm and render more accurate the opinion, which has been already determined from the rational symptoms, (and they ought therefore never to be omitted in the examination of such cases,) but these means are very seldom necessary to suggest the correct diagnosis originally. Three cases however are narrated by Laennec, in which the rational symptoms were obscure and very indistinct, and of which the true nature could not have been ascertained during life, had it not been for the assistance which was derived from auscultatory examination. The sound elicited by percussing the affected side of the thorax becomes unusually clear and resonant, in consequence of the escape of the air into the pleural cavity: the respiratory murmur is diminished, or altogether suspended; a blowing, or amphoric sound is in some cases heard to accompany each act of inspiration, and along with this sound the peculiar metallic tinkling also may be often perceptible. All these phenomena are however of uncertain occurrence; none is invariably uniform; and none therefore can be admitted as pathognomonic of the accident. The ruptured excavation may not at all, or may only imperfectly communicate with any of the bronchi; and under such circumstances, percussion does not avail, as no air has escaped into the bag of the pleura: and the amphoric sound and the metallic tinkling are also absent. The last-mentioned sign, the metallic tinkling, is in the majority of cases, not perceptible for several days after the accident has taken place, even although the ruptured cavity has a very free communication with the bronchi; and for this reason; that it is necessary to the development of this sound, that a certain quantity of fluid be present in the cavity of the pleura. Now this is seldom the case, while the contents of the vomica only have been effused; for these are in general too scanty. A degree of pleuritis being however immediately excited, the consequence of such an attack is an effusion of a sero-purulent fluid into the pleural cavity; and it is not until this period, that the metallic tinkling is heard. It is not easy to determine what quantity of fluid must be in the pleural cavity, before the tinkling can be perceived. Much may depend upon the consistence of the effused fluid, and probably also upon some other contingencies, which have not yet been well ascertained. In the history of perforation of the lung, perhaps no feature is more remarkable, than the great difference in the rapidity of its fatal progress, which different cases exhibit. Death took place sometimes in sixteen hours, and at other times, not for thirty-six days after the occurrence of the first declaratory symptoms.

This difference could not be satisfactorily accounted for, either by the relative strength of the patients, by the treatment adopted, by the size of the ruptured orifice, or by the quantity of fluid effused at the time of the accident into the pleural cavity.

"We insist," M. Louis very justly remarks, "on these details, because it is important that the physician should be aware, that in certain complications, mortal in their nature, the fatal termination may take place some hours or some weeks after their invasion, without his being able to explain on what these differences depend." 301.

Before closing these general remarks on this subject, we ought to notice the rapidity with which the effusion of turbid, or sanguineous fluid into the pleural cavity may take place, after perforation of the lung. It was considerable in one case, where death occurred in twenty-four hours, and where the sound on percussion during the first twelve hours was clear; and in another case, where the effusion was serous, the progress was equally rapid. These facts, however remarkable, are strictly in accordance with what is frequently observed in simple pleurisy, and more especially in the cure of hydrocele by injection, when a considerable effusion of purulent fluid is sometimes formed in the tunica vaginalis, in the course of a few hours.

We shall now present to our readers' attention, abridged reports of seven, out of eight of the cases of this fatal accident, which M. Louis has met with in his practice. They deserve to be studiously considered by every scientific physician, as affording the most accurate data for the recognition of the disease which have yet been published.

A man, aged 36, was admitted into the La Charité Hospital, on the 16th September. For five months previous, he had suffered, according to his own report, from cough, daily rigors, epigastric pain, and diarrhœa. Three days before his admission, he was attacked *suddenly*, after a fit of vomiting, with a severe pain in the left side, accompanied with dyspnœa and great anxiety, but these symptoms abating somewhat, he was able to walk to the hospital. On the 19th, the symptoms were acute pain in the side, breathing frequent and thoracic, orthopnœa, percussion unusually clear over the whole of the left side, where no respiratory murmur could be heard, or any metallic tinkling: intercostal spaces prominent and wide, cough rare and accompanied with but little expectoration, action of the heart scarcely audible. On the 5th of October, a confused murmur was perceptible in the upper fourth of the left lung, and opposite the inferior angle of the scapula; when the patient spoke a metallic tinkling could be heard. At this point and lower down, the sound on percussion was dull; anteriorly it was clearer than natural, but at this point there was no metallic tinkling audible: all pain had ceased. Until the 20th of the month, the metallic tinkling continued to be audible, not only at the original spot, but also over almost the whole of the left side posteriorly, under the axilla, and opposite the mamma. On the 20th it could not be heard anywhere. The patient remained constantly in the sitting posture, and on the following day he died.

*Dissection.* Nearly four pints of greenish-coloured pus were found in the left pleural cavity. Adhesions of the lung existed at several points. On the posterior surface of the lung, opposite the angle of the third rib, there was discovered "a rounded opening, four lines in diameter, the termination of a canal of the same dimensions, which, after an inch and a half, was continuous with one of the large bronchi. This canal was lined by a membrane, which reposed either on tuberculous granulations or healthy lung, and evidently resulted from a larger cavity, successively narrowed by the compression of the air and pus. There were some small incompletely excavated cavities in the summit of the same lung, with numerous grey semi-transparent granulations. The right lung presented superiorly a depression corresponding to a semi-cartilaginous mass, enveloped by a black and dry substance with some softened tubercles."

Tuberculous granulations and ulcerations were found in the lower fourth of the small intestines.

*Case* 2. A woman, aged 45, had laboured under the symptoms of phthisis for four months preceding her admission into the hospital. Auscultation detected the presence of an excavation in the upper lobe of the left lung; the pectoriloquy, cavernous respiration, and dulness on percussion were well marked. One morning, when free from suffering, she was attacked with pain near the inferior angle of the scapula, which was at first moderate, then *suddenly* very severe, accompanied with great anxiety and orthopnœa. The patient said she was suffocating: no posture was easy; the pain in the back was acute, and the slightest percussion on the chest insupportable. It is stated in the report that no metallic tinkling was to be heard; but the state of the patient must have prevented a satisfactory examination of the chest. She died two days afterwards.

*Dissection.* On opening the right side of the thorax, an inodorous gas escaped, and about four ounces of turbid fluid was found. The right lung was compressed to one-third its natural dimensions.

"Situated immediately below an adhesion posteriorly, there was a rounded opening, three lines in diameter, communicating with a small excavation, lined by a very thin false membrane, in contact with sound lung. This small cavity neither communicated with the bronchi, nor with a very large excavation just above it, which was invested by a double false membrane, one layer of which was soft, the other semi-cartilaginous. In the three lower fourths of this lung there were only some grey semi-transparent granulation. The left lung adhered to the costal pleura in its upper half, presenting at its summit a large excavation communicating with the bronchi, and also numerous small cavities; in the two upper thirds there were numerous grey granulations, surrounded by a yellowish, moist, semi-transparent substance, homogeneous, and entirely deprived of air; the remainder of the organ was red and hepatized." 283.

*Case* 3. The patient was 32 years of age, and exhibited all the symptoms of confirmed phthisis. Cavernous respiration, and doubtful pectoriloquy, might be heard between the shoulders and under both clavicles, and the patient complained of a sense of great heat at these parts.

"She was attacked by a *violent and sudden pain* along the vertebral column, accompanied with dyspnœa and anxiety. The next morning the patient was in the sitting posture, and spoke only of her pain and difficulty of breathing, expressing her surprise at the suddenness of the attack; the countenance was altered, and percussion clearer to the left, posteriorly and laterally, than on the right side. Over the same extent, instead of the respiratory murmur there was only a mucous ronchus, which appeared to traverse an empty space, before arriving at the ear; there was no metallic tinkling; and on the patient's lying down and rapidly rising, no peculiar sound was heard; the breathing was very frequent, with great agitation; she expired at 10 o'clock the same evening, after intense suffering." 284.

*Dissection.* "Only a small quantity of gas escaped from the left side, which contained about three pints of sanguinolent fluid, without any fragments of albumen. A soft membrane, of a deep red colour, and one-third of a line thick, everywhere invested the lung and thoracic parietes. The summit of the lung for two inches and a half was intimately adherent to the neighbouring parts; and almost immediately beneath this adhesion posteriorly, there was a rounded opening, about the size of a pea, communicating with a vast excavation, containing a very small quantity of a greyish fluid, similar to what was in contact with the diaphragm. This cavity communicated with the bronchi. The upper five-sixths of the lung were transformed into an indurated, greyish, semi-transparent substance, interspersed with numerous tubercles and small excavations; these communicated with each other, and in some points were only separated by a very thin layer, from the thoracic cavity; the lower sixth of the lung was crepitating; the bronchi were of a pale pink colour. There were some excavations in the summit of right lung, and at its base some crude tubercles; heart one-third less than its usual volume; aorta everywhere of a bright red." 285.

In the fourth case, the supervention of the *sudden* attack of pain, accompanied with great dyspnœa, and inability to lie down, and the unusually clear resonance of the chest over a large extent, where previously it had been indistinct, indicated the nature of the accident which had taken place, and that air had escaped into the cavity of the pleura. For the first four or five days, no metallic tinkling could be heard. It was then heard two inches below the clavicle, when the patient spoke. Subsequently, this peculiar auscultatory sound was perceptible over the lower three-fourths of the chest, which gave out a very clear sound on percussion. The patient died on the 18th day after the first occurrence of the symptoms.

*Dissection.* A quantity of gas escaped on opening the left side of the thorax, and a turbid greenish fluid was found in the cavity of the pleura. As in the former cases, the rupture was situated posteriorly, about two inches below the summit of the lung. It communicated with a large anfractuous vomica, which was pierced by the orifices of several bronchi. Both lungs presented numerous points of tubercular disease.

In the fifth case, the patient died on the second day after the *sudden* seizure, which indicated that rupture of the lung had probably taken place. On percussion, the left side, anteriorly, afforded a much more resonant sound than the right. In the same region, no respiratory murmur nor metallic tinkling could be heard.

"The upper lobe was enveloped by another false membrane, half a line thick, and semi-cartilaginous; and at its lower portion there was a rounded yellow patch, a line in diameter, corresponding to a softened tubercle, which had been partially discharged into the pleural cavity. The opening was in part closed by a small quantity of tuberculous matter, and the cavity lined by a thin, soft, light-coloured false membrane; there was no communication with the bronchi." 291.

In the sixth case, the patient died the same day that the accident took place. On dissection, an opening of two lines in diameter was found at the upper part of the left inferior lobe. It led directly to an excavation, which was lined by the remains of tuberculous matter, but without any false membrane, and which communicated with the bronchi.

In the seventh case, the patient survived the accident from the 26th to the 31st of December. The dyspnœa became *suddenly* very intense, amounting to orthopnœa; but there was no pain experienced, either at the time of the accident or after it, as had been the case in all the preceding six cases. Percussion on the left side was infinitely clearer than on the right; the respiration was confused, and, as it were, distant; and, after every respiratory movement, there was heard a sound similar to that produced by blowing into an empty bottle.

*Dissection.* In the left pleural sac, there was found a turbid reddish fluid, similar, except in density, to what is observed in tubercular excavations. The upper three-fifths of the left lung were adherent to the side; and, just below the line of adhesion, there was an opening, five lines in diameter, which was traced to communicate with a tuberculous cavity, and this cavity extended upwards into the interlobular fissure. Other cavities and depositions pervaded the substance of this, and of the right lung.

The preceding seven cases, with the addition of one more, are all the examples of the perforation of the lung, in connexion with tuberculous softening, which have occurred in M. Louis's experience. In five of these cases, the perforation was found at the same point, viz. opposite to the angle of the third or fourth ribs; and at this point the sudden pain had been felt. In seven of the cases, the lesion had taken place on the left side. This corresponds with an assertion formerly made by our author, that tubercular deposits are more frequent, and generally further advanced in development, in the left than in the right lung. On no occasion was there more than one perforation found. Often, indeed, there were observed vomicæ, which had reached the pulmonary pleura, and which would, in all probability, have burst into the pleural cavity, had the two surfaces of this membrane not been coherent at these points. This appearance is usually most evident towards the summit of the upper lobes, where the sides of the excavations are often solely formed by the false semicartilaginous membrane.

The tuberculous matter is sometimes found in immediate contact with the ribs, and occasionally it even traverses the intercostal muscles. In these examples, had there been no adhesions, it would have escaped into the pleural cavity, and the train of symptoms, which we have seen to characterize perforation of the lung, would have ensued. Here we must close our remarks on this very curious subject—a subject which hitherto has not attracted much attention. The account which Louis has given of the symptoms during life, and of the post-mortem appearances which accompany perforation of the lung, at least that form of it which is co-existent with, and dependent upon, tubercular lesions, is by far the most complete which has hitherto been published.

### Of the more marked Varieties of Phthisis.

Whoever has been much in the habit of attending to post-mortem examinations must be well aware, that a tuberculous state of one or of both lungs is not unfrequently met with in the bodies of patients, who had never, during life, exhibited any symptoms of pulmonary disease.* The existence of tubercles is, therefore, not necessarily associated with any very obvious disturbance of the general health. It is to this state that the term "latent phthisis" has been affixed by MM. Laennec and Louis; the germ of a fatal disease being not only present, but sometimes even considerably developed,

* Dr. Clark observes:—"Several of our celebrated pugilists have died tuberculous; and very lately one (Byrne, we believe) died of phthisis, a short time after an obstinately-contested fight, which proved fatal to his antagonist."

while its rational and more obvious signs or evidences are obscure, or perhaps entirely absent. If the question be put to a physician—what are the symptoms which would lead him to pronounce the existence of pulmonary consumption, he would very properly allude to the cough, expectoration of muco-purulent sputa, distress in the breathing, emaciation, hectic fever, and night sweats; and no one will deny that, if these symptoms be present, they would afford sufficient indication of the disease in question. Their absence, however, does not warrant the conclusion, that the lungs are exempt from serious tubercular injury. These organs may be the seat, not only of extensive tuberculous deposits, but even of numerous and large ulcerated cavities; and yet some of what are usually considered as the pathognomonic and necessary symptoms may be almost entirely absent. The cough, which, for the most part, is one of the earliest signs of pulmonary irritation, may be so unfrequent and so little troublesome, that the patient will deny that he ever coughs at all; and even his attendants may scarcely have noticed it. It may continue in this trifling degree for weeks, months, or even years, without any expectoration, and during all this time the lesion may be advancing, slowly and certainly, to its fatal termination. Every practical physician, who has seen many cases of phthisis, and who is in the habit, and has the skill, to examine with accuracy the state of the respiratory organs, by means of auscultation and percussion, must have met with instances, rare indeed, but still occasional, in which he has detected, even on his first visit, the existence of incurable disease of the lungs, when neither the patient nor his friends had the slightest apprehension on the subject.

We cannot be much surprised at this security, when we are aware that the disease may have arrived at its confirmed stage, without the manifestation of the more obvious symptoms, viz. the cough and expectoration. Portal long ago observed, "it is not sufficiently known that the disease can exist without the slightest cough; the lungs of consumptive patients have even been destroyed by suppuration, without their having experienced the least degree of cough." Now, although it is of very rare occurrence that the cough is absent during the entire period of the disease, we shall find, from the reports of some of the cases related by M. Louis, that it may be so trifling as to have escaped notice until within a fortnight of the patient's decease.

These few remarks, it will be understood, are intended merely as precautionary hints to the physician. They apply only to the exceptional cases of the disease; but the knowledge that such cases do occur, and that so prominent a symptom as the cough may be almost, or altogether absent throughout, ought to be impressed on the mind of every medical practitioner. What we have said of the cough is equally, and perhaps still more applicable to the expectoration. It does not belong to our present intention, to examine the various changes and differences in the characters of the sputa observed in the progress of phthisis. The transitions from the transparent, ropy, saliva-like mucus, to the admixture of the partially opaque, ash-coloured, rounded massules with this mucus, and, from this state, to the yellowish puriform state, are well known; and although, in the present days of improved diagnosis, physicians are not so busily anxious to discriminate between pus and mucus as they were wont to be in the days of our forefathers, the varying qualities of the sputa well deserve to be minutely studied. Chomel and Louis state that, in several hundred cases of pulmonary disease which they have accurately watched and reported, they have met with only two instances where the expectorated matter was streaked with more or less numerous yellow lines, and mixed, at first, with opaque whitish fragments, and afterwards with the rounded, globular, distinct masses, sometimes floating, and sometimes sinking in water, and yet in which the lungs were found, on dissection, free from tubercular disease. The affirmative value, therefore, of these appearances cannot be disputed; but let it be remembered, that extensive and most serious disorganization of the lungs may exist, while not only these appearances may be indistinctly traced in the sputa, but the very presence of any sputa whatsoever may have been entirely overlooked. That the sputa may be awanting, while the tubercles are still crude, we may, perhaps, not be surprised to hear; but that large and suppurating excavations may exist in one or both lungs, without any, or only very trifling expectoration having been present in any period of the disease, is a fact which may stagger the belief of the inexperienced physician. Portal has alluded to this anomaly—"Quelquefois ce crachement purulent n'a pas lieu, quoique les poumons soient pleins de foyers de suppuration;" and Dr. Clark alludes to "one decided case, in which the absence of expectoration continued to the last—the lungs, on one side, were found converted into a mass of tubercular disease, containing one largish, and numerous smaller vomicæ; and the upper part of the upper lung was similarly, but not so extensively affected." Several of Louis' cases illustrate the same fact.

Dyspnœa, variable indeed extremely in the frequency of its recurrence and in its intensity, is perhaps more uniformly present in phthisis than either the cough or the expectoration.* It is usually hurried on slight exertion—it becomes oppressed and panting, and very often shooting pains are felt, at the same time, through the chest or between the shoulders.

If this state of the breathing occur in a weak and delicate constitution, having any tendency to phthisical disease—if the patient be, at the same time, liable to flushes of feverish irritation after meals—if the cheeks, palms of the hands, or soles of the feet are apt to become hot, while the rest of the body is cool, or even chilly—if he loses flesh while he continues to enjoy his food, and takes it in the accustomed quantity, the suspicion of the medical attendant ought to be immediately aroused, and a full, patient, and most accurate examination of the local, as well as of the constitutional, symptoms be immediately instituted. The hereditary temperament and liabilities of the patient should be ascertained; particular enquiry should be made as to the presence of any morning cough and expectoration, and, if present, how long they have existed, and what causes appear to induce them: the breathing, the digestion, in short, the state of every function, ought to be rigidly scrutinized. The aids to be derived from percussion and auscultation are especially valuable, in the circumstances which we are now considering, and they ought on no account to be ever neglected. If the sound elicited by percussion, especially under the clavicles, be unusually dull, or if it is deci-

---

* We do not mean to say that this symptom is generally, at first, either very distressing to the patient, or very manifest to his attendants—more frequently it is not; but still the indications of a certain degree of annoyance or inconvenience in the breathing, may be always detected by the attentive physician.

dedly duller on one side than on the other, we have reason to suspect mischief lurking underneath; and if, upon applying the ear over the dull region, the respiratory murmur is less soft, easy, and slumbering than natural, or if it be altogether suspended, while it is louder, coarser, and more bronchial, and, perhaps, accompanied with a crepitating rhonchus in the neighbourhood of the mute region, and if the voice be, at the same time, more resonant there, our suspicions must be greatly confirmed. Whenever there exists a marked inequality in the sound elicited on percussion, and in the respiratory murmur, in the corresponding parts of the two sides of the chest, especially if these parts be the infra-clavicular, we have strong reason to apprehend tubercular disease of the lungs, unless such inequality can be otherwise satisfactorily accounted for. The combined value of the rational and physical signs is admirably described, in the following extract from Louis.

"When we therefore meet in the same individual, the *dry cough* which has existed a variable space of time, and in many instances come on without apparent cause, accompanied with *clear mucilaginous expectoration, pains* in the sides of chest or in the back, *hæmoptysis* from the commencement or during the progress of the cough, *dulness of sound under one or both clavicles,* diminution or any other alteration of the respiratory murmur in the same point, while the remainder of the lungs is healthy, *we may be certain of the presence of unsoftened tubercles.* The dyspnœa, the loss of appetite, the emaciation, the sensibility to cold, &c. which are present in this first period, *assist* our diagnosis, but could not, independently of the preceding symptoms, confirm it." 150.

Before adducing some cases, in illustration of the latent form of phthisis, we are tempted to give circulation to the following valuable remarks on a new auscultatory sign, contained in a note appended by Dr. Cowan.

"It may be here useful to mention another result of auscultation, on which the attention of medical men has only been lately fixed, and which was first signalised by Dr. Jackson, of Boston, in the wards of M. Louis, who has twice amply confirmed its value as an additional aid in diagnosis. We refer to the study of the *expiration.* In health this is scarcely and sometimes not at all sensible, and never seems to occupy the seat of inspiration, but is evidently at a distance from the surface, in the larger bronchi, and very feeble. But when the density of the lung is increased, the expiration becomes gradually more and more distinct and superficial, till it resembles a *second inspiration,* and frequently is alone heard; without accurate comparative examination, it might easily, and no doubt often has been mistaken, for the *inspiratory* murmur. What renders this sign peculiarly valuable is, that the change in the *expiration* precedes that of the *inspiration,* and consequently the modification is principally applicable to the early periods of the disease, where correct diagnosis is so important. I have frequently seen M. Louis, from this symptom alone, decide on the existence of induration of the lung, when it could neither be detected by percussion or modified inspiration." 150.

### Cases of Latent Phthisis.

*Case* 1. A man, aged 44, had been ailing for nine months before his admission into the hospital, on the 24th March. His chief complaints had been loss of appetite, thirst, frequent rigors, followed by heat and perspiration, weakness, and occasional pains between the shoulders. He had become much emaciated. There had been no cough, until within the six weeks previous to his admission. During the last three years, he had suffered several attacks of hæmoptysis. When received into the hospital, the sleep was found to be much interrupted by the cough; the sputa were yellow, greenish, but in separate masses, surrounded by a copious limpid fluid; cavernous respiration, with distinct pectoriloquy, was sensible between the shoulders and on the right side. He died on the 2nd of April. The summits of both lungs exhibited vast rugged excavations, which, from their walls being firm, resisting, and lined with a dense membrane, must have existed for several months at least; and numerous tubercles and granulations were dispersed through almost every part of both organs.

*Remarks.* The history of this case proves undoubtedly that extensive and most serious degeneration of both lungs may exist for a length of time, without having induced cough, or any strongly marked pectoral symptoms. The febrile malaise, and the decay of the general health and strength, which had so long preceded the occurrence of the cough, are no doubt attributable to the morbid changes which were going on all the time, in the lungs; and therefore these symptoms, alone, even when unaccompanied by any very obvious or distinct distress of respiration, should lead the physician at once to suspect the integrity of the lungs, in the case of a patient who had at any period of his life suffered an attack of hæmoptysis.

"Supposing for a moment that we had seen this patient soon after the commencement of the fever, we ought then, by means of the previous history, to have suspected the existence of tubercles in the lungs, and perhaps at this period, auscultation would have removed every doubt. We ought therefore *never* to neglect this method of investigation, whenever febrile symptoms are present without any evident cause, more especially if these have been preceded by one or more hæmoptyses." 241.

*Case* 2. A young woman, when admitted into La Charité stated that she had been subject to shortness of breath from her infancy, but that her present illness might be dated from about the two last years. During the first seven months, she had daily paroxysms of fever, and she lost her flesh and strength very rapidly. These feverish attacks gradually ceased, without any treatment having been used, and she then recovered, at least partially, her flesh and vigour. The usual dyspnœa however, was considerably increased, and *a cough, attended with expectoration, had commenced three months before her entrance into the hospital;* previously she had been quite free from it. The symptoms, rational, as well as auscultatory, announced to M. Louis, the existence of a large cavity in the summit of the left lung, From the time after admission, until her death, a period of four weeks, the state of the cough is thus alluded to, here and there, in the case book : "Cough principally violent in the morning; expectoration green, scanty and semi-opaque :" "cough little :" "cough occasionally violent :" "expectoration had ceased."

*Dissection.* A vast excavation in the upper left lobe; several small ones in the right. both lungs studded with tuberculous deposits.

*Remarks.* In this instance, tubercles had existed for a length of time, and even large excavations had been formed, before the cough had commenced. The febrile attacks experienced by the patient at the beginning of her protracted illness, are in our opinion to be attributed to the then incipient lesion of the lungs. Some may consider the disease to have been a simple ague; but the concomitant circumstances of the case are opposed to this idea. We have witnessed several cases in practice, where the early hectic or ephemeral fever which sometimes attends the primary deposition of tubercles, has been mistaken for a genuine intermittent, and has been treated accordingly, with most pernicious consequences. It cannot be too urgently recommended to the physician, that he should always examine most minutely and attentively the state of the respiratory organs, whenever the symptoms of ague shew themselves under what may be deemed a suspicious form, as for example, when the state of the patient's health has been for some time previously decaying, or when he has not been more than usual exposed to the agency of those causes which commonly induce agues.

*Case* 3. A woman, 31 years of age, delicate, and subject to shortness of breath from her infancy, complained of having been liable to frequent indisposition for several successive years. For the last five years she expectorated a little every morning, and during the first eighteen months of this period she had a constant cough. This however was never very inconvenient, and it ceased spontaneously, after a residence of some months at the sea coast. It was always excited by foggy weather, and by any strong scents. During the last three years, the usual dyspnœa had rather increased. She had suffered from stomach and liver complaints, and had been treated without advantage, by long courses of calomel, and purgatives. The catamenia had been generally very irregular, and sometimes absent altogether for several months. When admitted into the hospital, on the 2nd of January, the report states:—

"No dyspnœa when quiet, but it is brought on by the least exertion; no cough or expectoration; percussion everywhere clear; respiratory murmur natural, except under the right shoulder-blade, where it was stronger than in the corresponding point on the left side." 247.

At the end of February, there was slight cough, though the patient assured us that she did not cough at all; the breathing was quickened by the slightest movement, but from the almost entire absence of cough, we attributed this to the general weakness. The cough and expectoration were trifling, until within the last two or three weeks of her life. She remained nine months in the hospital, where she died on the 28th of September. On dissection, a large tuberculous excavation was found in the right upper lobe of the right lung, numerous smaller ones were scattered through the rest of this, as well as through the corresponding lobe on the left side.

*Remarks.* "During the nine months this patient was in the hospital, with the exception of the last fifteen days, it may almost be said, that *she had no cough*; and surely no one can doubt that the excavations in the lungs had existed for a long time anterior to this period." The development of the tubercular disease must have been extremely chronic in this case; for an attentive consideration of its history induced M. Louis to refer its commencement to between two and three years before its fatal issue. Appended to the report of the case is the following observation, which is interesting, as expressive of M. Louis' opinion, as to the curability of pulmonary consumption.

"The structure of the excavations also merit our attention. The largest was invested by a false membrane, lying upon almost healthy pulmonary tissue; this fact is rare, and it is only in analogous instances, *when the number of tubercles is small, that we can conceive the cure of phthisis to take place, by the cicatrisation of the excavation.*" 250.

In the fourth case, which occurred in a young female, 21 years of age, the absence of cough, until the last two weeks of her life, and the scantiness of expectoration, were perhaps still more remarkable. Up to the period of death, the sputa are described as "scanty, sometimes frothy and mucous, at other times completely opaque and flocculent."

The most prominent symptoms were oppression of the breathing upon any exertion, and an obstinately severe epigastric distress. Indistinct pectoriloquy, and cavernous respiration were audible between the scapulæ, extending principally to the right side. The summits of both lungs, were found on dissection, to be occupied with numerous tubercles, some of which were softened and excavated; and others were still crude. The peculiar softening of the mucous membrane of the stomach, to which we have alluded in the early part of this review, was very strikingly developed in the present case. To this, we must ascribe the severe and protracted suffering in the region of the epigastrium, which this patient so long complained of.

The fifth case is very similar to the preceding one, in respect to the indistinctness of the pectoral, and the very marked prominence of the gastric symptoms, viz. the pain in the region of the stomach, the appetite variable, or uniformly deficient, the nausea, and frequent vomitings.

From the commencement of the disease in May, to and on the 26th of August, the day of her death, *there was no cough*, with the exception of the last ten days; it was then attributed by the patient to her throwing off the bed-clothes during the night; it excited little attention, and on account of her weak state, auscultation was not practised. The most prominent symptoms were occasional difficulty of breathing, repeated febrile attacks, and severe stomach distress.

*Dissection.* The upper lobe of the right lung was sprinkled with innumerable miliary grey semi-transparent granulations, more or less opaque in their centre. In the corresponding lobe of the left side, there was a middle-sized tuberculous excavation, lined by a semi-cartilaginous membrane.

These five cases, with the addition of other three, have been adduced by M. Louis, as examples of what he calls "latent phthisis;" a form of the disease peculiarly distressing, from the insidious progress of the evil, before its very existence may have been suspected.

The proportion of such cases, to those of what may be called the regular form of phthisis, is, according to the experience of M. Louis, about one to fifteen. If however the hæmoptysis, which so often precedes the cough and expectoration, is to be considered in the majority of instances the effect, rather than as a precursory symptom of tubercles, the relative frequency of latent phthisis must be stated much higher. With respect to the causes, or influences, which may tend to mask and conceal the existence of tuberculous disease, in that form of phthisis which we have been attempting to illustrate, it is certainly not easy to arrive at any very definite conclusions. The age of the patient might be supposed to have some influence; but our data are neither sufficiently numerous, nor accurate to warrant us in stating, at what period of life it is most apt to occur. The ages of the eight patients whose cases are recorded by M. Louis, were 82, 44, 22, 31, 21, 24, 62, and 19.

It is reasonable to suppose, that the constitutional disposition or hereditary tendency to consumption, has been generally either not very strongly marked, or that it has been counteracted by peculiarly favourable circumstances, as of climate, occupation, &c.; and, as far as we can determine from the reports of Louis' cases, the remark holds true of them. But, perhaps, of all causes, the most frequent and the most influential is the cotemporaneous existence of some other morbid process, or it may be of pregnancy (for this condition seems to have the same effect), in the system. The observations of Dr. Clark on this subject are excellent, and cannot be curtailed without disadvantage.

"Tuberculous disease is rendered latent, or is at least masked by a peculiar condition of the system in some cases; and in others by the presence of other diseases. Pregnancy appears to retard if not to suspend the progress of phthisis, and it is frequently observed that the disease advances with great rapidity immediately after parturition. The catamenia generally cease when the disease has made some progress; although they continue in a few rare cases until death. An attack of mania in a phthisical patient has been followed by the suspension of the pulmonary disease; which, however, rarely fails to carry off the patient ultimately, whether the attack of mania has ceased or not. The complication of dyspepsia with tuberculous disease is not an infrequent cause of the latter being overlooked, the dyspeptic symptoms being more evident than the phthisical. The aspect of the patient in such cases is pale and unhealthy; he gets thinner and weaker; the food which he takes neither affords him nourishment nor strength; and yet he has no evident ailment but what is referrible to the deranged state of the digestive organs. In such cases there may be no cough, no fever nor expectoration to excite our fears for the safety of the patient; while at the same time tubercles are present in the lungs. We have seen a patient of this kind, when asked any question respecting the state of his lungs, strike his chest, and confidently affirm that all was right there; although his lungs were tuberculous to a considerable extent at the time. This is the form of the disease which has been termed 'dyspeptic phthisis.'

Diarrhœa is another disease which sometimes disguises phthisis, and its effects in suspending all the usual symptoms of pulmonary affection are often remarkable. We have known more than one example of extensive tuberculous disease of the lungs being detected on dissection, when the cause of death has been looked for in the intestines. It is true that these were cases in which the early history of the disease was disregarded, but they serve at least to shew the power of diarrhœa in masking extensive affections of the lungs." 19.

### ACUTE FORM OF PHTHISIS.

The duration of tubercular phthisis varies from a few weeks to twenty years and upwards. Perhaps the average length of time, reckoning from the earliest softening of any of the tubercles to the period of death, may be stated to range from nine to eighteen months. In the very rapidly fatal cases, those to which, in this country, we apply the term "galloping consumption," the phases of the disease so quickly succeed each other, that the vital energies appear to be at once overpowered, and not even a favorable remission of the symptoms is at any time observed. From the first open establishment and manifestation of the mischief, its progress is sure, speedy, and appalling; and the young practitioner is sometimes apt to suppose, that he may be treating a case rather of an acute bronchitis, or pneumonia, than one of genuine tubercular consumption. M. Andral has reported four examples of this rapid form of phthisis, which varied in duration, from twenty-one to thirty-five days; and, according to the table given by M. Louis, it appears that six of his cases proved fatal from the 24th to the 80th day. Dr. Clark has described the characters of acute phthisis with his usual ability.

"The acute form of phthisis admits of a useful division into two varieties, in one of which the short duration of the disease appears to depend chiefly on its violence, or the activity of the morbid process; and in the other, on the feeble powers of the constitution, which sink under the pulmonary disease long before it has reached the degree in which it usually proves fatal. This last variety is observed chiefly in delicate young persons, and more frequently, according to our observation, in females than in males. The ordinary state of health of such persons is far below the common standard; they are possessed of the highest degree of the tuberculous constitution or temperament; they are weak, easily fatigued, and have generally a languid circulation, indicated by a feeble pulse and cold extremities, even in their best health, and before any suspicion exists that these general phenomena are connected with tuberculous cachexia."

"There is a slight cough, with some shortness of breathing; and the pulse is frequent, or easily rendered so by the slightest exertion. The patient is weak, but scarcely considers herself ill; there is no pain of chest, no hemoptysis, and perhaps no expectoration. Debility is usually considered the cause of these symptoms, and even when they are accompanied with morning perspirations and well-marked emaciation, the friends are scarcely alarmed. They tell us that she was always short-breathed and liable to cold; and the cough seems of so little consequence, that they think the lungs must be sound. In this way the patient continues to become rapidly worse; the cough is more troublesome, and is by degrees accompanied with some expectoration, in which a tinge of blood occasionally appears. The breathing is now also observed to be quick, even when the patient is at rest; the pulse is rapid, and there are frequent and often very copious morning perspirations. The countenance of the patient alone, at this time, is very often sufficient to indicate the danger: it is generally pale and of a leaden hue, the lips are often of a blueish colour, and the albuginea of a peculiar dull pearly tint; the whole features are sunk and the countenance faded. Without any increased activity of the symptoms, such a patient may sink rapidly under an attack of diarrhœa, and a fainting fit unexpectedly terminate the scene."

"The other variety of acute phthisis presents itself in more striking characters. All the symptoms of phthisis are present in an unusual degree of severity, and succeed each other with great rapidity. The cough increases daily, and the expectoration goes through its various changes in the course of a few weeks; the hectic fever is violent, the morning perspirations are copious, and diarrhœa usually contributes its share in the destruction of the patient." 12.

The *acute* form of pulmonary consumption is generally observed in young persons, and especially after the cessation of acute febrile diseases, as fever, scarlatina, measles, &c. or of an attack of hæmoptysis. Most of the cases which have occurred in our own practice have been the direct consequence, we might even say the continuation, of a sudden and violent attack of spitting of blood. The patients never recovered, and sometimes never left their beds since the invasion. It is not improbable, that the very remedial means which may have been deemed necessary to arrest the frightful hæmorrhage have, by reducing the strength of the system, and, by causing a general re-action (so often the effect of large and repeated bleedings), tended to accelerate the changes of the tubercular deposits. Catarrh and pneumonia may have the same effect. As a matter of course, the tubercular disease of the lungs is, we must suppose, already established; and it only requires the application of some cause inducing pulmonary congestion or inflammation, while the system is at the same moment in a state of great debility, either from protracted indisposition, or from temporary excessive evacuations, to give, as it were, the momentum to the process of disorganization. The doctrine involved in the following paragraph of Dr. Clark's treatise appears to us of very questionable accuracy.—"The rapid progress of phthisis often occurs in persons of a constitution so highly tuberculous, that it only requires the application of some exciting cause to *determine the deposition* of tuberculous matter in the lungs." We think it much more probable that there has been a tuberculous disease of the lungs latent for a considerable time previous to the invasion of the acute symptoms, at least in those cases which prove fatal in the course of a few weeks. It is our duty, however, to state, that the opinion of Dr. Clark is countenanced by, and, indeed, derived from, M. Louis, who, in the observations affixed to the second case of acute phthisis, remarks—

"It cannot be supposed that when the fever and dyspnœa commenced, that tubercles already existed in the lungs; for before this the patient was *in perfect health,* and it is difficult to imagine such numerous granulations to be present without impeding the pulmonary functions; so that everything indicates that tubercular deposition was here extremely rapid." 266.

The following examples, abridged from Louis' reports, will form a useful commentary on the preceding remarks.

*Case* 1. A girl, aged 18, of a moderately-strong constitution, was admitted into La Charité on the 29th April. She had been little subject to colds, usually in good health, and dated her present illness as of only fifteen days' standing. It commenced by rigors, followed by flushes of heat and by perspiration. These attacks became more frequent and of longer duration; they induced complete loss of appetite, general malaise, and very considerable debility. On the tenth day from the commencement, there was slight cough and expectoration observed for the first time. These symptoms were, in the course of a few days, much aggravated, the cough becoming very frequent and troublesome, the respiration thoracic and much hurried, and the sputa semi-opaque and greenish. Pains were felt under the sternum and left clavicle. The feverish irritation of the system, denoted by rapid pulse, intense heat, red tongue, great thirst, anorexia, and headache, continued. From this period till the 16th day of May, the progress of the disease was rapid and continuous, the expectoration becoming more decidedly phthisical, the breathing more distressed and quickened, varying from 45 to 60 times in the minute, and the auscultatory signs more distinct and expressive. The report of the 16th states, that the pulse has become gradually more and more frequent; heat much elevated and dry: thirst very urgent, the patient drinking five or six pints of fluid in the 24 hours; constant night-sweats, &c. Death took place on the 19th.

*Dissection.* "The left lung offered some adhesions posteriorly; the upper lobe contained numerous grey semi-transparent granulations, surrounded by slightly engorged pulmonary tissue; the engorgement was more considerable in the base of the lower lobe, which contained a few granulations; the right lung, universally adherent, was transformed at its base into a mass of tuberculous matter of a pale rose tint, to the extent of two inches in height and two in breadth, occupying nearly the whole circumference of this part of the lung; it was perforated by a kind of anfractuous canal, enclosing a small quantity of a thick dark coloured fluid. Elsewhere the lung presented numerous semi-transparent granulations, and small softened masses of tuberculous matter: its tissue was slightly engorged." 262.

The abdominal viscera were nearly quite sound.

*Remarks.*—" In this observation the interval between health and disease was short; the cessation of one, and the commencement of the other, were well marked; the duration of the disease was thirty-five days, that of the cough twenty-five. The intense nature of the symptoms is as remarkable as the rapid progress of the affection. At first, intense febrile movement, associated after ten days with cough, expectoration, and dyspnœa; these rapidly increased; from the sixth day of the cough, the breathing was 47, and still more accelerated on the succeeding days; the temperature was much elevated, and pulse frequent; all pointed out an affection of the lungs." 262.

M. Louis adds; " it is remarkable, that notwithstanding the extreme rapidity of tuberculous development in the preceding case, there were scarcely any traces of inflammation in the surrounding textures, not even in the right lung, which, we supposed from the symptoms, during life, would have been chiefly affected." From this he reasons, that tubercular changes are not necessarily of an inflammatory character, as Broussais has maintained.

*Case* 2. A young man, 19 years of age, was admitted into the hospital on the 4th of May. He stated that he had been ill about twenty days; that his illness commenced with cough, clear expectoration, and general feverishness; that these symptoms gradually increased in severity, and became accompanied with flying pains through the chest, and with headache.

The day after his admission, it is reported:—

" Face hot, red, animated; eyes brilliant, lively; headache, with dorsal decubitus; oppression moderate, little cough, expectoration like frothy saliva. On the left side there was pain under the edge of the false ribs, and percussion was dull in the lower half the same side of chest posteriorly, and anteriorly under the clavicle; cavernous ronchus very distinct in the latter region, and opposite the shoulder there was incomplete œgophonia." 267.

During the last eight days of his life, the dyspnœa was urgent and the breathing very rapid. The pulse was always accelerated, generally as high as 100, and sometimes even much quicker. The heat elevated, the thirst very urgent, the night perspirations copious, and these were followed by occasional rigors. The cavernous ronchus and respiratory noise became more and more distinct, especially under the left clavicle, but without pectoriloquy. Percussion was dull on the left side below the mamma, and over two inferior thirds of the chest. He died on the 4th of June.

*Dissection.*—" Left lung everywhere adherent, by means of dense resisting false membrane. At its summit there was a half emptied cavity of about the size of a nut, with numerous tubercles, and some isolated hepatized portions of lung; the remainder of the whole lung was almost entirely converted into tuberculous matter, disposed in masses of variable dimensions, between which there was scarcely a tenth of the parenchyma permeable to the air. The right lung was free, with numerous tubercles at its summit, many of which were softened or half excavated; they were confined to this portion of the lung; the thin edges of the upper and middle lobe were hepatized." 268.

*Remarks.* If we date the commencement of the disease in the preceding case from the invasion of cough and fever, (and before this period there had been no functional derangement), its total duration may be reckoned at fifty days. It is not easy to explain the presence of the œgophony heard in the early stage of the case.

In the third case, which proved fatal in between four and five weeks, the affection commenced without any evident cause, and directly after the patient had partaken of a moderate repast, with rigors, followed by heat and perspiration. After the first four-and-twenty hours, the breathing became oppressed, the dyspnœa continually increased, and there was occasional cough. The febrile attacks gradually became more frequent and severe; and the strength of the patient very rapidly decayed. The morbid appearances in the lungs are thus described.

"Their tissue was red, and granulated over the greater part of their extent, easily broken down, especially on the right side; firmer at the summit than the base, yielding when pressed, a thick dark coloured fluid, which was mingled with a little air inferiorly; there were numerous grey, semi-transparent granulations, diminishing from above downwards. They were opaque and yellowish in their centre, varying in size from hemp to that of millet seeds, the latter of which were without the central opaque spot." 265.

Thus we have detailed at length the post mortem appearances, discovered in three of the cases of acute, or rapidly fatal phthisis, in order that the reader may be enabled to recognize the differences in their character and extent, from those which are usually found after protracted chronic cases of the disease. It will be perceived that the middle and lower lobes of the lungs are comparatively more frequently affected in cases of the acute form; and that the whole of one lung is sometimes found almost completely infiltrated with tuberculous deposit, in various stages of development, in one part consisting chiefly of the grey semi-transparent matter, in another of the genuine yellow tuberculous matter. When vomicæ are found, they are, as might have been predicted, small, irregular, incompletely excavated, with walls which are soft, yielding, and not lined with any distinct membranous coating, but merely with a thin layer of semi-fluid lymph.

On the contrary, when vomicæ have existed for a considerable length of time their parietes are almost constantly more or less hard and resisting, and are formed either of the yellow tuberculous matter, of the grey substance, or sometimes of melanotic matter. The membrane which lines an old excavation, is dense, greyish, almost semicartilaginous, and is generally covered by another membrane of very slight consistence, of a yellowish, or whitish colour, and usually distributed in patches. Sometimes there is no lining membrane at all; and then, the pulmonary texture, more or less considerably altered, is uncovered, forming the parietes of the cavity. The contents of an old vomica are very often of a greyish, or greenish tint, having a dirty and disagreeable appearance, thin, of moderate consistence, and sometimes tinged with blood, or even of a deep red colour. Their smell is occasionally extremely offensive and putrid, resembling that of animal substances, which have been macerating for some time. The contents of a recent vomica have usually the character of common purulent matter. Moreover an old excavation is generally more uneven and rugged, than one that is more recent. It is frequently crossed in different directions by cord-like bands, or intersections, formed by the grey semi-transparent matter, which is interspersed with genuine tubercles, and in which blood-vessels, either obstructed or open, are sometimes, although rarely, found. When the softening of the deposited tuberculous matter takes place rapidly, as in acute phthisis, a large extent of one or both lungs, and sometimes even the entire lung of one side, undergoes the morbid change simultaneously, becoming soft, and easily broken down throughout the whole of its substance. At different parts of such a disorganised mass, and more especially towards the summits of the lungs, one or more irregular cavities may be found, more or less filled with puriform matter, but not exhibiting the firm lining membrane, nor the intersecting bands, which are observed in vomicæ of longer standing. Such are some of the most striking differences in the morbid appearances noticed after acute, from those observed after the regular or chronic form of the disease. It is unnecessary to allude to the marked differences in their symptoms during life. These are too obvious and characteristic to escape the attention of the least exact. Not so, however, is the line of demarcation between the acute form of phthisis, and another form recognized by Dr. Clark, and to which he has affixed the term *febrile*, "from the degree of fever with which it is usually ushered in and attended during its whole course." In order that our readers may, by comparing Dr. Clark's description, with the reports of the cases of acute phthisis, which we have already extracted from Louis, judge for themselves of the propriety of establishing another form of the disease, we shall give the description of it in Dr. C.'s own words.

"The attack of febrile phthisis is generally sudden, occurring in a state of apparent health, after exposure to cold, or even without any very evident cause. We say apparent health, because we believe that the disease never occurs in a healthy constitution. It attacks persons of a tuberculous diathesis; and the most marked cases which have come under our observation have occurred in persons having a strong hereditary disposition to phthisis,—in members of families, several of whom had already fallen victims to the disease in its usual form.

It commences with shivering, followed by heat of skin, quick pulse, and the other symptoms of fever, which often continue for several days with little or no indications of pulmonary disease. In some cases it puts on the characters of bilious fever, and in others of catarrhal fever, for both of which it is sometimes mistaken; indeed we believe it would have been such in a healthy constitution, but occurring in a person labouring under tuberculous cachexia, the rapid deposition of tuberculous matter in the lungs is the consequence of the disturbance created in the system by the febrile attack. Cough, however, soon appears, and the breathing is noticed to be particularly rapid, which is one of the most marked and constant symptoms of this form of phthisis. The cough, when it has once occurred, becomes speedily more frequent, and is soon accompanied with some expectoration, which is at first colourless, afterwards assuming a yellowish or greenish hue, and occasionally being streaked with blood; but it rarely puts on the character of the expectoration in the advanced stages of ordinary phthisis. Pain of one or both sides frequently occurs, and occasionally diarrhœa is present. The fever, in the mean while, continues without abatement, and is so much out of proportion to the other symptoms of pulmonary affection, that the true character of the disease is liable to be overlooked. In the course of from three to six or seven weeks the patient sinks." 17.

Now what is the short, but comprehensive description of acute phthisis, given by Louis.

" The number of our observations is too limited to justify any general description of acute phthisis, or to allow us accurately to delineate its diagnostic symptoms. We think however this form of the disease ought to be dreaded, in those cases where dyspnœa, cough, expectoration, fever, with sometimes pain of chest and very hurried respiration, come on suddenly and without evident cause." 273.

And if the reader will take the trouble to read attentively the three preceding cases of acute phthisis, he will find, we think, their symptomatology, and that of Dr. Clark's febrile phthisis to be very much alike. The "particularly rapid" breathing which is considered by Dr. Clark, as "one of the most marked and constant symptoms of febrile phthisis," was very conspicuous in the majority of the cases. It is particularly alluded to in the first report, in which the respiration is stated as " varying from 49 to even 60 times in the minute." The circumstance too of the expectorated matter not exhibiting all the characters it usually presents in regular phthisis, is well illustrated in the report of the third case, where "the dyspnœa and breathing were rapid the last eight days; the cough was sometimes violent, but usually moderate; and the expectoration became *partially greenish, but not striated*, towards the last." This case proved fatal in seven weeks from the commencement of the phthisical symptoms.

Dr. Clark then proceeds to notice the occasional difficulty of a correct diagnosis in this form of phthisis, which, he tells us, may be mistaken for acute bronchitis, or even for pneumonia; a mistake, which is the more excusable as the tuberculous disease in its progress is often complicated with one or other of these affections.

But is not the same difficulty, and this too proceeding from the same cause, experienced in some cases of the acute form of the disease? Dr. C. himself has in a previous part of his treatise very justly observed—

" The error into which this variety of acute phthisis is calculated to lead an inexperienced or careless practitioner, is that of considering and treating it as a purely inflammatory disease, and using much more active measures, and giving a more favourable prognosis, than the real nature of the case justifies. An inquiry into the previous health of the patient and careful observation of the symptoms will soon unveil the real nature of such cases. It is true that inflammation in some part of the respiratory organs often exists, complicating the tuberculous disease; but it requires to be treated with much more delicacy than a simple inflammation, and a very different prognosis should be given." 13.

The pathology of febrile phthisis is stated by Dr. C. to be "somewhat peculiar." The morbid appearances generally observed, "consist of the grey granulations over a greater or less extent of the lungs in some cases; in others, large portions of the lungs appear to be converted into a mass of cheesy-like tuberculous matter, the pulmonary tissue being completely infiltrated with it. The tuberculous matter is less frequently confined to the summits of the lungs in this form of phthisis than in any other. Tuberculous cavities are also found in some cases, but they are generally of small size, only partially evacuated, and have no lining membrane, as occurs in cavities of long duration in the ordinary form of phthisis." Now, no better description could be given of the pathology of acute phthisis, at least of Dr. Clark's second variety of this form, than that here detailed, which we have transferred from his to our own pages. Having said so much as to the general symptoms, and the pathological characters of *febrile* phthisis, it must be unnecessary to allude to the auscultatory signs which are said to assist in discriminating it from the other forms of the disease. They may be easily inferred from a knowledge of the state of lungs usually found on dissection. The dull sound elicited by percussion is heard over a large extent of one, or of both sides of the chest. The respiratory murmur is louder, coarser, and more bronchial than usual, and is frequently accompanied by a mucous or crepitating râle. Pectoriloquy, when present, is usually imperfect, and the metallic tinkling sound is seldom or perhaps never heard. This description is equally applicable to the acute, as to the febrile form of phthisis.

For the reasons now adduced, we are unwilling to admit the *febrile*, as a distinct form of the disease, and we think it probable that Dr. Clark, on a revision of the subject, will come to the same conclusion. Some physicians have maintained that most cases of acute and febrile phthisis are strictly and essentially of an inflammatory character. The experience of Louis, and the reasoning of Dr. Clark, prove that this explanation is highly improbable. An attack of pneumonia or of pleuritis may, and very often does, supervene during the course, and more especially towards the close of ordinary phthisis, and the latter disease will then assume all the fatal rapidity of a genuine acute case. The knowledge of this fact is very necessary to the practical physician, as the symptoms of the inflammatory attack are sometimes so faint and obscure, that they are apt to be overlooked. All accessions of high feverish irritation in a phthisical case ought therefore to be attentively watched; not indeed that every such attack indicates pulmonic inflammation, (for we have already seen that pyrexia will be induced by the mere rapid evolution of tubercles in the lungs,) but only because such an attack is by no means of improbable occurrence. Indeed, during the course of other chronic diseases, as well as of phthisis, a similar, but not so great a tendency to pulmonic and pleuritic inflammation is frequently observed. M. Louis states that, in 112 cases where death took place during the last stage of chronic diseases of various sorts, in twelve he found a portion of one, and sometimes of both lungs, red, granulated, and hepatized, and in other ten cases a high degree of congestion in these organs. The result of his observation is, that "tubercles and tubercular excavations are nearly without influence over the development of pneumonia in the last stage of phthisis." The same remark is applicable to pleuritis; a disease which is often developed in the last period of phthisis, and also of other chronic maladies; arising sometimes from evident causes, as the application of cold to the surface of the body, but most frequently without any appreciable cause. The converse of these propositions is also stoutly maintained by our author. His work is full of data adduced to prove that pneumonia and pleuritis exert but little influence, except so far as they weaken and injure the general health, on the development of phthisis. Among other arguments, he alludes to the well-established facts, that pneumonia is most usually developed from the base to the summit of the lungs, while the reverse is the case with tubercular disease; that pneumonia seldom attack both sides of the chest, while phthisis almost invariably occupies both lungs; and that pneumonia is more frequent in men than in women, the reverse holding good with phthisis.

We shall close our remarks on this subject by the following passage from Dr. Clark, with whose opinion we entirely coincide.

"We believe that inflammation in a tuberculous constitution may give rise to the deposition of tuberculous matter in place of lymph, which is its usual product in healthy subjects, and this may be one of the sources of tuberculous disease; but it does not follow that it is the most constant or even most frequent cause of tuberculous deposits. A striking objection also occurs to this view of the case, in the fact that pneumonic inflammation is no infrequent occurrence during the progress of the disease, and that this is characterized during life by its proper symptoms, and after death by the usual morbid appearances; one part of the lungs presenting the usual results of pneumonia, while in others we find the tuberculous infiltration which has just been noticed." 18.

Bayle, Laennec, Andral, all agree in regarding pneumonia as a very secondary cause of phthisis. The contrary opinion, so obstinately held by Broussais, is contradicted by unprejudiced experience.

So much of our space has been occupied with the consideration of the *latent* and *acute* forms of pulmonary phthisis, that we can afford but little room for any remarks on that form, which, from its very slow progress and protracted duration, has been denominated lingering or *chronic* consumption. We have already stated, that the average duration of phthisis may be considered to be from nine months to a year and a half, or two years. But this period is occasionally prolonged to a continuance of many years. According to a table of 114 cases, the duration of each of which was determined as accurately as possible, it appears that, in 19, the disease lasted from two to twenty years, giving thus the proportion of rather less than a fifth of the whole number. These 19 cases are arranged as follows:

| Duration of the Disease. | Number of Deaths. |
|---|---|
| $2\frac{1}{2}$ | 2 |
| 3 | 4 |
| 4 | 6 |
| 5 | 2 |
| 10 | 1 |
| 12 | 2 |
| 14 | 1 |
| 20 | 1 |

Chronic phthisis occurs more frequently in patients of from twenty-five to forty years and upwards, than at an earlier period of life. The tubercular predisposition, we may suppose, is either never very strong, perhaps not at all hereditary, and, therefore, accidentally induced or acquired, or it has been kept in check, and counteracted, by the favourable circumstances in which the patient has been placed. Thus a change of climate, when as yet no signs of the disease have ever shewn themselves, is not unfrequently observed to preserve one member of a family, all the rest of whom may have been swept away. We have often had occasion to remark this in the case of sons, who had been sent out in their youth to the East Indies, and had remained there for a number of years.

The approaches of chronic phthisis are generally most insidious and obscure. A slight and occasional cough, which attracts little attention, and which ceases altogether on the setting in of fine weather—a general weakness, and incapacity for long-continued exertion—a disposition to stomachcomplaints; these symptoms, from being trifling in degree, and, perhaps, only of short duration at a time, are perhaps altogether overlooked, or attributed to a mere common catarrh, or to an occasional dyspepsia. A visit to the country in Summer may restore the patient to an apparent perfect health. But, in the succeeding Winter, the cough is found to return, and to be easily aggravated by all changes in the weather; perhaps the breathing becomes hurried and oppressed on any exertion, and then the skin is apt to be bedewed with perspiration; the patient complains of fits of chilliness coming over him, without any evident cause; the hands, and more especially the feet, are with difficulty kept comfortably warm; the cough is usually more troublesome in the morning when he first awakes, and it induces scanty expectoration of frothy, saliva-like mucus, colourless and uniform at first, but afterwards more stringy, having intermixed with it specks of opaque matter, which partly sink and partly float in water, in the form of striæ. A slight attack of hæmoptysis is not unfrequent at this period of the disease.

Such may be the state of the patient during the Winter and Spring months. As the warm and genial weather commences, all the troublesome symptoms may gradually cease—the cough may leave him—his flesh and strength return, and he may appear to be capable of strong and frequent exertion. Generally, however, the cough is apt to return every now and then, as after sudden changes of the weather, or any severe exertion of the lungs, as in speaking, singing, &c.; the appetite may be very good, but the patient remains thinner than might have been expected, and he finds that he is obliged to cease bodily, or even merely mental labour sooner than his inclination would lead to do.

"This is a state of things (says Dr. Clark) which is not uncommon in persons living in easy circumstances, and who are not required to make much exertion, or expose themselves to the vicissitudes of the weather, or to other exciting causes. They are considered delicate; they find it necessary to take care of themselves, but the nature of their ailments frequently remains long unsuspected. The cough is little regarded, because it does not increase and gives very slight trouble, and even abates so much during the summer as to be scarcely remarked. The breathing is short, but the dyspnœa has come on so slowly that the patient is hardly aware that it is a new complaint, and often deceives himself in thinking that he was always short-breathed. There is often among consumptive persons a jealousy of being interrogated on any symptom which seems connected with pulmonary disease; and they not uncommonly conceal such symptoms from the physician, who must, if he desires to arrive at the truth, put his questions with great caution, and without appearing to attach any importance to them. Invalids of this description are rarely free from dyspepsia in a greater or less degree; they are liable to an increase of the catarrhal symptoms from slight exposure to cold, and are frequently subject to attacks of diarrhœa, and from which their recovery is often tedious and protracted." 14.

Year after year may pass over, and the patient may, with care, enjoy moderately good health; should he, however, be seized with any inflammatory disease, especially of the respiratory organs, or with any smart febrile attack, the tuberculous disorder is apt to be immediately roused to activity, and the patient may rapidly sink under it, with all the symptoms of a well-marked consumption, which is then very generally attributed, both by himself and his attendants, to the invasion of what was merely a superadded and accidental occurrence. Unfortunately we are obliged to confess, that even the physician often allows himself to be deceived as to the true state of things.

There is the less excuse for this in the present day, when the diagnostic signs have been made so much more exact and expressive, than they were before the discovery of percussion and auscultation. We cannot too urgently call upon all our professional brethren, never to omit to examine the chests of their patients, in every case of thoracic complaint, whether it is attended with suspicious circumstances, or not. Such men as Andral, Louis and Chomel, not to mention a host of others, would not have been so unanimous in their praises of auscultation, had experience not taught them its paramount utility. The extent and degree of degeneration sometimes found in the lungs of those who have died of chronic phthisis, are truly astonishing. The entire lung on one side and more than a half of the other one, are not unfrequently mere masses of tuberculous deposit in its various stages of crudity, and softening, exhibiting in some parts one or two vast excavations, and in others, numerous smaller ones.

The last form of phthisis to which we shall allude derives its name and peculiarity of character, from the early period of life at which it occurs, and from the seat of the chief lesion discoverable on dissection. When tubercular disease attacks infants and young children, the deposition is found more frequently, in the substance of the bronchial glands, than of any other portion of the thoracic contents; and hence the term *bronchial* phthisis has been proposed to denominate this form. M. Lombard has given two tables in his ingenious Essay on Tubercles, with the view of exhibiting the relative frequency of the disease in the different organs and viscera of the body, at different periods of life.

In 100 adult phthisical patients, he found, (not to mention the lungs, which were as a matter of course diseased in all) tubercles in the intestines 26 times; in the mesenteric glands, 19 times; in the bronchial glands, 9 times; in the spleen, 6 times; in the brain, and its envelopes, 3 times; and in the kidneys and some other internal viscera only once; whereas in the same number of cases of bronchial phthisis, the proportional frequency is shewn by the following figures:—The lungs themselves, 73—intestines, 9—mesenteric glands, 31—bronchial glands, 87—spleen, 25—brain and its envelopes, 15—and kidneys, 11.

Our confidence in the accuracy of these tables is inhanced by their results coinciding with the investigations on the same subject, made by M. Papavoine, who has published an excellent memoir in the 2nd vol. of the Journal de Progrès des Sciences Medicales. The researches also of Louis, Andral and Alison, all tend to the same general conclusion, that the proportional frequency of tubercles in different organs varies much in the infant and adult. In the former, they usually exist in a greater number of organs at once, and they are not so invariably present in the lungs.* Their more frequent occurrence in the brain and its meninges, in the spleen and kidneys, but above all, in the bronchial glands, are facts of curious interest to

* Louis, it appears, is not quite willing to admit that tubercles are so frequently, if indeed they are ever, found in the other viscera, and organs of the body, even in the case of infants, while the lungs are totally exempt from them. He says; "we have never observed tubercles in a single instance in any organ, without their existing in the lungs at the same time; so that their presence in these last viscera, seems a necessary condition for their development in other parts." Andral, Lombard, &c. differ from Louis, and assure us that they have occasionally discovered tubercles in various organs, when none were to be observed in the practical physician, as well as to the pathologist. It is in the bronchial glands, that the tubercular phthisis of young children usually commences. The disease in some cases does not extend beyond them, and yet will prove fatal, without affecting the lungs, or any other organ.

The following case, derived from Dr. Alison's paper on Scrofula, in the Transactions of the Edinburgh Medical Society, is an instructive example.

" J. S. æt. five, a boy of ordinary stature, and pretty stout, but somewhat rickety, and with a small scrofulous sore on his leg, was attacked in the end of November 1815, with well-marked pneumonic symptoms. While these were recent, he was seen by different medical men, who had no doubt of their nature, and he was bled twice at the arm, and used the other usual remedies, with very imperfect success: the heat of skin, febrile oppression, and dyspnœa abated somewhat, but his breathing continued short, his cough very troublesome and dry; and he passed gradually into the state of perfect hectic, the rigors in the afternoons and morning sweats being unusually severe. He died, considerably emaciated, in the end of January 1816. On dissection, the lungs were found of the natural spongy texture throughout, and the disease appeared to have been confined to the bronchial glands, which were enormously enlarged, and all converted into the usual cheesy or tubercular matter. There was no other disease in thorax or abdomen." 16.

In general however, tuberculous disease does not remain long limited to the bronchial glands: the lungs first, and then the mesenteric glands are gradually involved in the morbid process, and the train of symptoms becomes consequently more and more complicated. The bronchial membrane, in the neighbourhood of the diseased glands, is usually but not always found in a state of inflammation, and no doubt for the same reason, that when the mesenteric glands are tuberculous, the adjoining portion of intestine is commonly more or less deeply inflamed.

The symptoms of bronchial phthisis are usually at first very obscure and unsatisfactory; and the disease may continue long, and may have advanced to a most serious extent, before its presence has been even suspected.

The child is afflicted with an obstinate catarrh, remittent in severity, but never entirely absent for a length of time: the cough is in many cases paroxysmal, recurring in fits, as in pertussis: it is rarely attended with expectoration, until the latter stages of the disease, and even then this last-mentioned symptom may appear to be entirely wanting, from the sputa being swallowed. The breathing is hurried and distressed upon every exertion. The child usually points to the upper part of the sternum, as the seat of its chief distress. Hæmoptysis is of very rare occurrence in infantile phthisis. The hectic fever is never so perfectly formed, nor the perspirations so profuse, as in phthisis in the adult subject: the emaciation however, and decay of the system may proceed with very fatal rapidity. When the symptoms now enumerated occur in a young child, and when, upon a minute examination, neither pulmonic, nor mesenteric disease can be distinctly inferred,

---

the lungs. In reference to the opinions of these authors, Dr. Cowan has very sensibly remarked: " We are disposed to think that the *latter fact* is not satisfactorily established, either for infants or adults, and that the value of M. Louis' observations on this point (which only present one exception in 350 examples), is not yet impaired, for we must remember that M. M. Andral and Lombard, not considering the grey semi-transparent granulations to be a modification of tubercles, they no doubt have omitted to mention them in their calculation, and this may be the cause of the non-accordance of their results with those of M. Louis." 120.

we may justly suspect, that there is tuberculous disease of the bronchial glands. Our suspicions will be confirmed, if our patient be at all predisposed to a tuberculous diathesis from hereditary taint. The form of consumption, which we have been considering, is far more common than hitherto has been generally believed. Dr. Guersent assures us that, at the Hôpital des Enfans in Paris, five-sixths of those who die in that establishment, exhibit on dissection, traces of more or less extensive tuberculous disease. What the proportion is of these cases, which can be considered examples of phthisis, the data do not warrant us to say positively. The progress of bronchial phthisis is usually slower than that of the genuine pulmonary form, and the prognosis is certainly more favorable, in the former case, than in the latter; the organs primarily affected being less essential to life than the lungs.

"The termination of this disease is various. That the tuberculous state of the bronchial glands may be removed by absorption, as we see occur in the lymphatic glands of the neck, we have every reason to believe; but this is probably the less frequent termination. Another mode of cure is that by which the softened tuberculous gland empties itself into the bronchial tube with which it is in contact, by ulcerative absorption of the walls of the tube, as is shown in Dr. Carswell's beautiful plates. The matter being evacuated, the cavity in which it was contained gradually contracts till it is obliterated; and the cure, as far as this gland is concerned, is complete. The less frequent cure is that in which a portion of the gland, or rather of the tuberculous matter, remains in a cretaceous form."—*Clark*, 16.

The comprehensive survey which we have taken of the pathological characters, and of the more remarkable varieties of phthisis has occupied so much more space than we contemplated, that for the present we must close this article.

To discuss the subject of its treatment may engage our attention ere long. We have laid the foundation for sound and rational, and we may even add, successful practice, by pointing out distinctly what is the enemy we have to encounter, the warnings of its approach, its hidden and insidious attacks, the development of its movements, and the numerous and complicated machinery with which, as it advances, it assails the bulwark of life, and with which it finally obtains the inevitable victory of death. There is not a position in a physician's career, which requires the exercise of greater professional tact, of more collected, yet kind and humane feelings, of more unshaken self-possession, of more soothing pity, and of a more perfect knowledge of the resources of his art, what it can do, and what it cannot, and what it ought therefore not to attempt, than when he is called upon to attend one, in whom the early symptoms of pulmonary consumption have manifested themselves. How much depends upon the diagnostic decision which is then formed? If the physician can confidently and unhesitatingly satisfy himself of the true nature of the case from the beginning, how many weeks, and months, and even years of most perplexing anxiety, will be spared to himself, as well as to the poor sufferer! All our rational hopes of a successful treatment must be limited to the first stage of the disease; and when we are told of cures upon cures of confirmed consumption, we much fear, that the proclaimers of such success, are, if not willing impostors on the credulity of the public, mischievous dupes of their own ignorance. Independently of the serious, and too often irreparable mischief inflicted on the structure of the lungs themselves, the widely extended dissemination of tuberculous deposits, in other structures and in other organs, must forbid any hopes of a cure in the advanced stages of the disease. M. Louis' Researches have shewn us that in a large majority of the fatal cases of phthisis, the stomach and more especially the intestines, the liver, and spleen, the various groupes of lymphatic glands, &c. are all deeply involved in the morbid change. As well then might we hope to tear a lusty tree from its bed, by dividing one only of its many roots, as to *cure* a case of pulmonary consumption, when the system is penetrated with the multitudinous evil.

Such vain endeavours are fraught only with reproaches against the quackery of the medical art, and with needless sufferings to the patient, on whom they are tried. A far more worthy object of useful and creditable exertion presents itself to the enlightened physician, in foretelling the threatened approaches of the deadly evil, in counteracting its early symptoms, and in the steady employment of those means, which are best calculated to arrest its progress.

In our next number we shall probably return to this subject, and bestow upon it that consideration which its importance demands.

Our opinion of the high merits of M. Louis' researches must be apparent from the attention with which we have treated them. To Dr. Cowan the thanks of the profession are due for having naturalised one of the most valuable productions of the foreign press, a work to which constant appeal and reference will be made by all future writers on phthisis.

